# Genome-wide DNA methylation dynamics at “heading” stage of panicle and flag leaf in contrasting rice cultivars under field drought conditions

**DOI:** 10.3389/fpls.2025.1707950

**Published:** 2025-11-11

**Authors:** Ringyao Jajo, Shivani Kansal, Saloni Mathur, Saurabh Raghuvanshi

**Affiliations:** 1 Department of Plant Molecular Biology, University of Delhi, New Delhi, Delhi, India; 2 BRIC-National Institute of Plant Genome Research, New Delhi, Delhi, India

**Keywords:** methylation, bisulphite sequencing, *Oryza sativa*, rice, drought, N22, IR64, stress

## Abstract

**Introduction:**

Drought stress induces widespread genome-wide alterations in DNA methylation of rice. These changes work to alter gene expression and are relatively unexplored in reproductive tissues like flag leaf and panicle under field drought conditions. This study aims to explore the same in the panicle and flag leaf tissue of IR64 (drought-sensitive) and N22 (drought-tolerant) rice cultivars under field-drought conditions during the ‘heading’ stage of development.

**Methods:**

For the same, we generated whole-genome bisulfite sequencing libraries from the corresponding tissues and analysed them in detail.

**Results and discussion:**

The DNA methylation dynamics in adult tissue (flowering stage) was found to be clearly distinct from that of the seedling stage. Further, the contrasting rice genotypes also exhibited cultivar-specific and drought-induced dynamism in the methylation signatures. Notably, the two cultivars demonstrate inherent distinctions in sequence preferences of hyper- and hypo-methylation even prior to experiencing drought stress, and these preferences persist under the influence of the stress. Approximately 90% of the drought-induced differentially methylated region (DMR) are cultivar-specific, and about 70% of the cultivar differences (cultivar-DMR) under stress are unique compared to control condition. There is higher prevalence of hyper-methylated DMR that co-localized with differentially expressed genes in panicle. DMR of CHH sequence exhibit stronger negative correlation with expression compared to CpG and CHG sequence. Examination of differentially expressed genes with DMR highlights their functional relevance under drought stress, especially with DMR found in gene bodies and promoter regions. Notably, in panicle, methylation divergence of the two cultivars influences flowering regulation genes. Additionally, the findings also suggest a regulatory role for DNA methylation in drought induced response of miRNA genes, particularly in the panicle of N22 cultivars.

## Introduction

Rice (*Oryza sativa* L.) is an important food crop consumed by over half of the global population. It is considered a lifeblood of the Asia-Pacific region (Papademetriou et al., 2000). The varying ability of rice cultivars to adapt to drought stress presents significant potential to identify molecular determinants for potential crop improvement. Rice cultivars IR64 and N22 are two such contrasting cultivars: IR64 is well recognized and cultivated for its high quality but is sensitive to drought stress, whereas N22 is known for its drought tolerance (Lafitte et al., 2007; Wade et al., 1999; Reddy et al., 2009; Jagadish et al., 2008; Mackill and Khush, 2018).

The dynamism of DNA methylation patterns in plants is an important mechanism that regulates gene expression in response to environmental stimuli. It is an epigenetic modification where a methyl group is covalently added to cytosine bases of DNA sequence to form 5-methylcytosine. In plants, methylation occurs in all three cytosine contexts, viz., CpG, CHG, and CHH, where H represents A, T, and C. The maintenance of the three contexts involves different regulatory mechanisms. METHYLTRANSFERASE 1 is involved in maintaining CpG (Zhang et al., 2018; Kankel et al., 2003). CHG methylation is catalyzed by CHROMOMETHYLASE 3 (CMT3) and to a lesser extent by CMT2 (Lindroth et al., 2001; Zhang et al., 2018; Stroud et al., 2013). The maintenance of CHH methylation involves different catalytic enzymes depending on genomic location. DOMAINS REARRANGED METHYLASE 2 mediated by RNA-directed DNA methylation (RdDM) maintained CHH methylation in euchromatic regions and long transposons, whereas in heterochromatin regions, where RdDM pathways are inhibited, it is maintained by CMT2 (Zemach et al., 2013; Zhang et al., 2018).

Drought stress induces extensive genome-wide alterations in DNA methylation in rice (W., S., Wang et al., 2011; Rajkumar et al., 2020; Garg et al., 2015; Wang et al., 2016; Kou et al., 2021; Zheng et al., 2013; Wang et al., 2011; Gayacharan and Joel, 2013). The distinct differences in drought tolerance characteristics of tolerant and sensitive rice cultivars have led to investigations into their varying responses in methylation (Garg et al., 2015; Rajkumar et al., 2020; Wang et al., 2016, Wang et al., 2011). These studies have emphasized differences in methylation patterns between tolerant and sensitive cultivars under stress. However, uncertainty remains whether such differences exist even under non-stress conditions and the extent to which these variations persist under stress conditions. Moreover, the studies have predominantly focused on the methylation response of seedlings or under greenhouse conditions. It is anticipated that the fundamental methylation profile could be notably influenced by factors such as plant developmental stages, tissues, and growth conditions. The evidence from other studies has shown that DNA methylation gradually increases and that the variation decreases with developmental stages in plants (Zluvova et al., 2001; Bitonti et al., 2002; Ruiz-García et al., 2005). Furthermore, unlike in the greenhouse, plants in the field are exposed to multiple factors that affect the developmental phase. Thus, this warrants a more detailed investigation of methylation dynamics under the field environment at the reproductive stage of rice. Consequently, the current study aimed to comprehensively characterize genome-wide cytosine methylation landscapes in the panicle and flag leaf tissues of drought-sensitive (IR64) and drought-tolerant (N22) rice cultivars grown under field conditions and to elucidate the extent and nature of methylation changes induced by drought stress during the “heading” stage of rice. The study also aimed to examine the inherent differences in methylation between the contrasting genotypes under a non-stress environment. Furthermore, the role of DNA methylation in regulating the expression dynamics of miRNA genes during drought stress was also explored. The results reveal distinct sequence preferences for methylation between IR64 and N22, and the pattern persisted even under drought stress conditions. It underscored the regulatory impact of DNA methylation on gene expression in a sequence-context dependent manner, exhibiting notable variation in strength among the CpG, CHG, and CHH contexts. Collectively, these findings provide a comprehensive understanding of the epigenetic mechanisms underlying drought tolerance in rice.

## Materials and methods

### Plant growth and drought stress treatment

*O. sativa* L. subsp. *indica* cv. IR64 and N22 were the two rice cultivars used in the study. The two cultivars were grown in different beds at the Division of Genetics, IARI, New Delhi, as previously described (Balyan et al., 2017). Briefly, planting fields were set up to mimic the field conditions, and necessary care was taken to prevent water seepage in and out of the field. Drought was simulated by withholding water supply in individual beds, 10 days prior to the expected mean “heading” date of IR64 (110 days after sowing) and N22 (90 days after sowing) to achieve a soil moisture content level of 15%. The “heading stage” is defined by the full exertion of the panicle from the leaf sheath.

### DNA isolation and whole genome bisulfite sequencing

The libraries were prepared from snap-frozen tissues of the panicle and flag leaf harvested from the field-grown plant and stored at −80 °C. The EpiGenome™ Methyl-seq Kit was used for preparing the whole-genome bisulfite libraries. Prior to the EpiGenome kit procedure, DNA samples were treated using the EZ DNA Methylation-Gold Kit (Zymo Research, California, USA), and the resultant DNA was used to prepare the libraries in accordance with the manufacturer’s protocol. For each tissue of IR64 and N22, two biological replicates were utilized in the analysis.

### Determining methylated cytosines

The bisulfite reads were trimmed and mapped to the reference rice genome (MSU v7.0) using CLC Genomics Workbench (v9.0.1). Only unique mapped reads were considered for analysis. For further analysis, cytosines with at least 5 read depths per base were used to call the methylation level. The error rate was determined by comparing the data from the chloroplast genome (Greaves et al., 2012). The error rate ranged from 1.39% to 1.44% (**Supplementary Table S1**). The error rate in computing the binomial distribution was used to determine the true methylated cytosines. Cytosines were considered methylated if *p*-value ≤ 0.005 and present in both the biological replicates. Genomic regions corresponding with the methylated sites were annotated using ChIPseeker (v1.26.2) (Yu et al., 2015).

### Determining differentially methylated regions

The significance of the differences between the methylated regions of a 100-bp non-overlapping window was determined using Fisher’s exact test (*p* < 0.01) and False Discovery Rate (FDR)-adjusted *p*-value (<0.05). The MethylKit package (v1.22.0) of R was used to determine the non-overlapping regions (Akalin et al., 2012). The regions with at least five cytosines and having a methylation level variance of >1.5-fold change were considered as true differentially methylated regions (DMRs).

### RNA-seq data

The RNA-seq data were used, which were prepared and analyzed in earlier studies from our laboratory (Gour et al., 2022). In brief, paired-end libraries were prepared using the TruSeq RNA Library Prep Kit (Illumina, Inc., California USA) and the Illumina Novaseq platform. In this study, genes were classified into silent, low, medium, and high based on the average Fragments Per Kilobase of transcript per Million mapped reads (FPKM) of the three biological replicates under stress. Silent genes were defined as those with an average FPKM of less than 0.5 in drought stress. Genes with an average FPKM of up to the second quartile were classified as having low expression levels, genes in the third quartile as having medium expression levels, and genes in the fourth quartile as having high expression levels. For determining differentially expressed genes, genes with FPKM ≥ 0.5 and a significance threshold of FDR *p*-value ≤ 0.05 with a twofold change were considered.

### MiRNA data analysis

The bisulfite-seq data generated as above were also used to analyze methylation patterns with reference to miRNA genes in rice. The genomic coordinates for different features of all the miRNAs were calculated based on the coordinates of the precursor region taken from miRbase v.21. The different features were i) mature miRNA; ii) precursor miRNA; iii) promoter up to 3 kb, divided into three equal parts, upstream of precursor start site; and iv) downstream up to 3 kb, divided into three equal parts, downstream of precursor end site. Using these features as “regions of interest”, differential methylation for each feature was calculated using the MethylKit package (v1.22.0). The heatmaps were drawn using “ggplot2-v3.3.6”. The degradome predicted targets of miRNAs were compiled from in-house-generated and analyzed data (Mutum et al., 2016) and from the PmiREN database (Guo et al., 2022). The targets passing the criteria of *p*-value ≤ 0.05 and category 0–2 for in-house-generated data, or category 0–2 and degradome evidence of at least four libraries from the PmiREN database were selected.

## Results

### Global methylation profile of drought-sensitive (IR64) and drought-tolerant (N22) rice cultivars under control conditions

**Figure 1:**
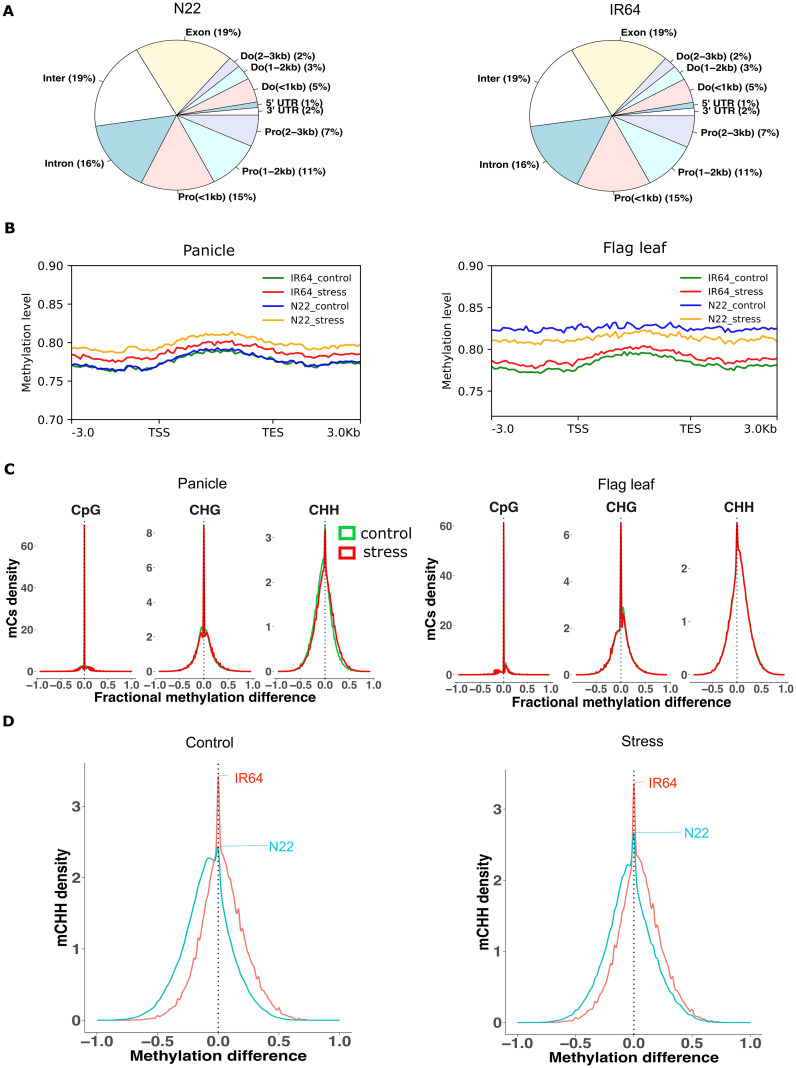
Methylation profile of IR64 and N22. **(A)** Distribution of methylated cytosines across different gene regions in the panicle. Pro, promoter; Do, downstream; Inter, distal intergenic. **(B)** Methylation level across different gene regions from 3-kb upstream to 3-kb downstream regions. TSS, transcription start site; TES, transcription end site. **(C)** Kernel density graph of methylation difference between N22 and. IR64. Greater than zero indicates hyper-methylation, and less than zero indicates hypo-methylation in N22 compared to IR64. Green and red colors represent control and drought conditions, respectively. **(D)** Kernel density plot of methylation difference of CHH between panicle and flag leaf in IR64 and N22 under control and drought stress. Greater than zero indicates hyper-methylation in panicle compared to flag leaf and vice versa.

In the panicle, approximately 9% in IR64 and 10% in N22 of the total genome cytosines were found to be methylated under control conditions. In both cultivars, the share of the CpG context accounted for approximately half (48% in IR64 and 49% in N22) of the total methylated cytosines (mCs), followed by CHG (29% in IR64 and 30% in N22) and CHH (23% in IR64 and 21% in N22) (**Supplementary Tables S1**, **S2**). A significant proportion of methylated sites were concentrated in the gene body (exon and intron; 35% in both cultivars) as opposed to the distal intergenic region (19% in both cultivars) (**Figure 1A**). The methylation levels across different gene regions were similar between the cultivars (**Figure 1B**).

A comparison of overlapping cytosines in the panicle between cultivars at single-base resolution under control conditions revealed that more than 60% of methylated sites were conserved (70% in IR64 and 64% in N22) (**Supplementary Table S3**). The majority of these overlapped cytosines were of the CpG context (52%), followed by CHG (31%) and CHH (17%). The methylation levels of the overlapped mCs differed only slightly, with the tolerant cultivar (N22) exhibiting marginally lower methylation levels in the CHH context (**Figure 1C**). The methylation composition and distribution pattern of the flag leaf were similar to those of the panicle (**Supplementary Table S2**, **Supplementary Figure S1**). However, in the flag leaf, the methylation level across the gene regions of N22 was slightly higher than that of IR64 (**Figure 1B**). Approximately 78% of the total mCs found in the flag leaf of N22 overlapped with IR64 (**Supplementary Table S3**). Unlike in the panicle, the shoulder on the right of the CHH contexts in **Figure 1C** indicates that the tolerant cultivar (N22) tended to have a higher methylation level than IR64. When comparing the flag leaf data with seedling data from a previous study (Garg et al., 2015), we observed notable variation. The overlap in methylated cytosines between the two developmental stages accounted for less than 30% (29% in IR64 and 25% in N22) (**Supplementary Table S4**). Additionally, methylation levels appeared to be higher in mature tissue (**Supplementary Figure S2**).

The analysis of mCs between tissues under control conditions also revealed that a large proportion of flag leaf overlapped with the panicle in IR64 (~83%) and N22 (~90%) (**Supplementary Table S5**). More than half (54%) of the tissue-overlapped mCs were composed of CpG in both cultivars. Comparing the methylation levels of the two tissues revealed that the flag leaf tended to have a higher methylation level of CHH compared to the panicle in N22, but the differences were less noticeable in IR64 (**Figure 1D**; **Supplementary Figure S3**).

The results indicated a high degree of conservation in DNA methylation patterns between IR64 and N22 under control conditions, particularly at CpG sites. Nevertheless, subtle differences were evident—most notably, the drought-tolerant N22 exhibited slightly lower CHH methylation in the panicle but higher methylation in the flag leaf compared to IR64.

### Differentially methylated regions of IR64 and N22 under control conditions

**Figure 2:**
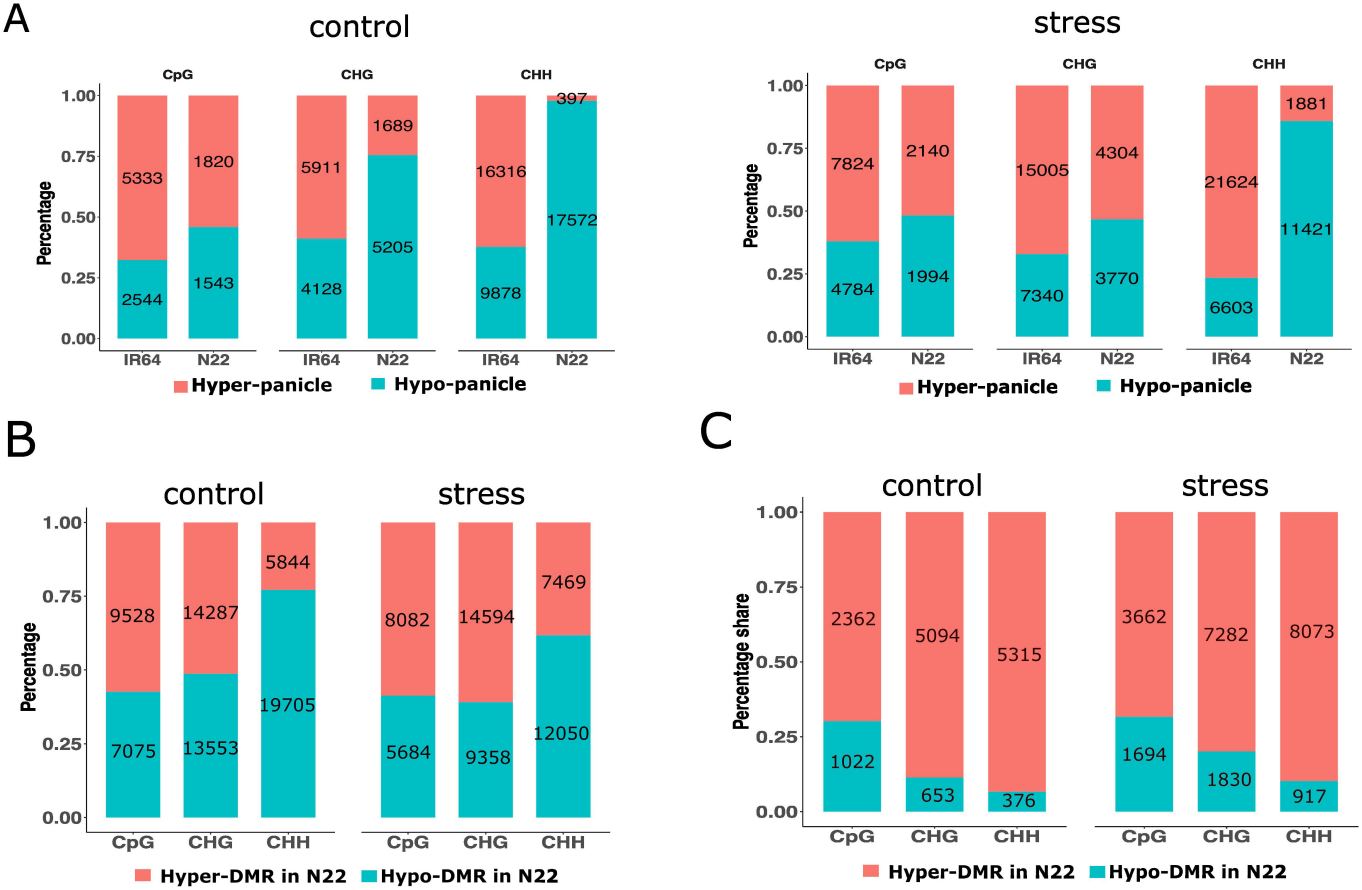
Profile of hyper- and hypo-methylated DMRs of tissues and cultivars. **(A)** Percentage share of hyper- and hypo-methylated tissue-DMRs under control and drought stress. **(B, C)** Percentage share of hyper- and hypo-methylated cultivar-DMRs (in N22 compared to IR64) in panicle **(B)** and flag leaf **(C)**. Number in the bar indicates the count of tissue-DMRs or cultivar-DMRs. DMRs, differentially methylated regions.

To further analyze the differences between tissues under control conditions, DMRs between panicle and flag leaf were identified within 3-kb upstream and downstream regions of the gene in both cultivars. The total number of tissue-DMRs (panicle *vs*. flag leaf) found in IR64 and N22 was 44,110 and 28,226, respectively. The majority of these tissue-DMRs (59% in IR64 and 64% in N22) were composed of CHH, while the least constituted CpG contexts (18% in IR64 and 12% in N22). Therefore, a strong association was observed between tissue-DMRs and cytosine contexts (CpG, CHG, and CHH) in both cultivars, indicating a sequence-dependent methylation pattern (IR64: Cramer’s V = 0.555, *X*^2^ = 13629, *p* < 0.0001; N22: Cramer’s V = 0.661, *X*^2^ = 12345, *p* < 0.0001). In IR64, 62% of tissue-DMRs were hyper-methylated in the panicle, compared to only 14% in N22. The association of hyper- or hypo-methylation and cytosine contexts was strong in N22 but weak in IR64 (N22: Cramer’s V = 0.51, 95% CI [0.49, 0.52]; *X*^2^ = 7271, *p* < 0.001; IR64: Cramer’s V = 0.057, 95% CI [0.045, 0.068]; *X*^2^ = 148, *p* < 0.001). Under control conditions, tissue-DMRs in N22 were preferentially hypo-methylated in CHH (90%) and CHG (70%) (**Figure 2A**).

The differences between N22 and IR64 under control conditions were also investigated, and DMRs between the cultivars were analyzed for both tissues. A total of 69,992 DMRs were detected in the panicle, hereafter referred to as cultivar-DMRs (cDMRs). In the panicle, under control conditions, cDMRs were predominantly in the CHG (40%) and CHH (36%) contexts. Cultivar-DMRs were further classified as hyper- or hypo-methylated in N22 relative to IR64, termed *hyper-cDMR* or *hypo-cDMR* in N22, respectively. The analysis of cDMR status indicated a preference for a specific sequence context for hyper- or hypo-methylated cDMRs. This association was moderate in strength (Cramer’s V = 0.30, 95% CI [0.29, 0.31]; *X*^2^ = 6423, *p* < 0.001). In N22, CHH sites showed a higher proportion of hypo-cDMRs, while CpG and CHG displayed nearly equal proportions of hyper- and hypo-cDMRs (**Figure 2B**).

Furthermore, a total of 14,822 cDMRs were identified in the flag leaf. Approximately 77% of the cDMRs were in the CHG (39%) and CHH (38%) contexts. The association of hyper- or hypo-methylation of cDMRs on sequence contexts was weak in the tissue (Cramer’s V = 0.26, 95% CI [0.24, 0.28]; *X*^2^ = 1039, *p* < 0.001). Nevertheless, more than 65% of cDMRs were hyper-methylated in N22, indicating that hyper-methylation predominated regardless of cytosine context (**Figure 2C**).

### Methylome landscape of rice under drought stress

In the panicle under drought stress, genome-wide methylation levels increased in IR64 and N22 (**Figure 1B**). In contrast, the flag leaf exhibited increased methylation in IR64, but a decrease in N22 (**Figure 1B**). The percentage share of methylation contexts across the gene regions remained consistent (**Supplementary Figure S4**). The pairwise comparison of mCs between control and drought conditions revealed that ~70% of the mCs identified under control conditions overlapped with those under drought stress in both tissues of the two cultivars (**Supplementary Table S6**). Likewise, comparative analysis between the cultivars under stress showed that over 61% of the methylated cytosines in N22 overlapped with IR64 in the panicle, with approximately 90% of these shared sites overlapping under control conditions. Methylation dynamics between the cultivars under stress were more pronounced in the CHH context as compared with the other contexts (**Figure 1C**). Higher methylation levels tend to be associated with IR64 in the CHH context as compared to the tolerant cultivar. In the flag leaf, over 70% of mCs in N22 overlapped with IR64, and 78% of these overlapping sites were consistently maintained under control conditions. The contextual composition of overlapped mCs in the flag leaf mirrored that observed in the panicle. However, the tolerant cultivar tended to exhibit higher methylation levels in the CHH context compared to the drought-sensitive cultivar (**Figure 1C**).

**Figure 3:**
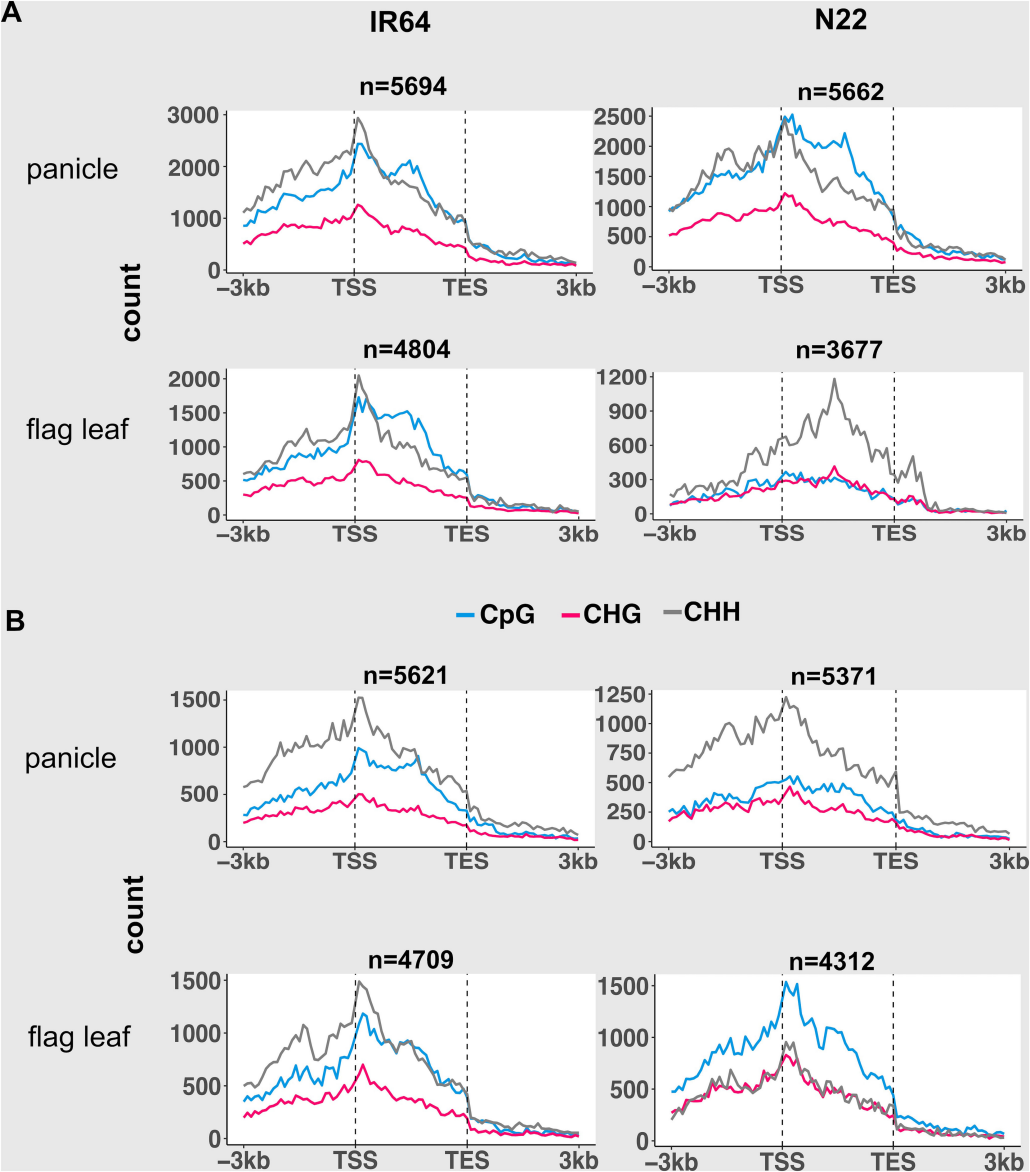
Abundance of demethylation **(A)** and *de novo* methylation **(B)** of CpG, CHG, and CHH across gene regions under the drought stress condition, shown only for genes with high expression categories (above third quartile of expression). Gene body is converted to proportion. Dotted vertical lines represent the alignment of transcription start site (TSS) and transcription end site (TES). n indicates the number of genes.

Under drought stress, up to 30% of the total mCs in both tissues of IR64 and N22 were demethylated (DI), while *de novo* methylation (DII) accounted for 12% to 59% of the total mCs (**Supplementary Table S7**). In the panicle, N22 exhibited a distinct preference for CpG demethylation within the gene body of expressed gene categories, whereas IR64 showed preferential CHH demethylation in the promoter and near the TSS region (**Figure 3A**; **Supplementary Figure S5**). Conversely, DII in both cultivars was enriched in CHH within promoter and gene regions, with N22 showing conspicuously higher levels (**Figure 3B**; **Supplementary Figure S6**). In IR64, the gene body also showed a high preference for CpG sequence. The abundance of DI and DII across gene regions of the flag leaf and panicle was similar in IR64. In the flag leaf of N22, CHH demethylation (DI) was predominant across gene regions and increased with gene expression, while *de novo* methylation favored the CpG context across the regions in all the gene expression categories. Interestingly, over 54% of DI- and DII-associated genes overlapped between cultivars (**Supplementary Table S8**), indicating substantial shared targets but genotype-specific sequence preferences that shape methylation dynamics.

Notably, in the two dynamicity of methylation, the silent gene category exhibited a distinct pattern compared with other expressed gene categories, and the pattern was stably maintained across the genomes and tissues (**Supplementary Figures S5**, **S6**). There were noticeable peaks in promoter regions close to TSS and downstream regions close to the transcription end site (TES) of silent genes.

### Differentially methylated regions of IR64 and N22 under drought stress

**Figure 4:**
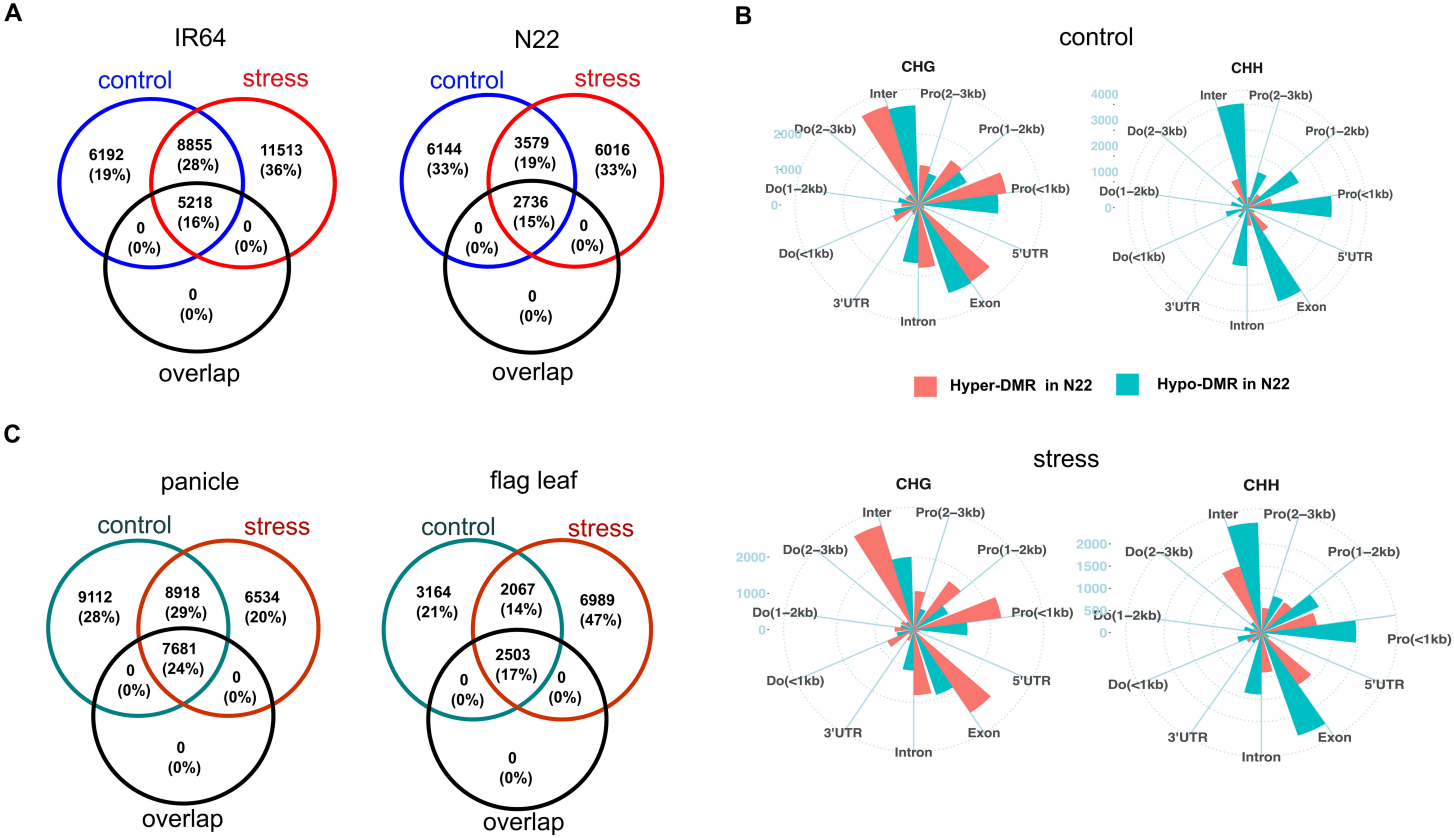
Comparison of DMRs between control and drought stress. **(A)** Venn diagram representing the number of genes with tissue-DMRs that overlapped between control (blue circle) and stress (red circle). “Overlap” (black circle) represents the number of genes whose tissue-DMRs overlapped between control and drought stress. **(B)** Distribution of hyper- and hypo-cDMRs across gene regions of panicle. Pro, promoter; Do, downstream; inter, distal intergenic. **(C)** Venn diagram of overlapped genes with cultivar-DMRs that overlapped between control (green circle) and stress (red circle). “Overlap” (black circle) represents the number of genes whose cDMRs overlapped between the growth conditions. Number in the bracket **(A, C)** represents the percentage of genes in each category out of the total genes found in both control and stress. DMRs, differentially methylated regions.

We further investigated the tissue-DMRs under drought stress in both cultivars. In IR64, the number of tissue-DMRs was higher under stress as compared to the control condition, whereas N22 exhibited fewer tissue-DMRs under stress. Statistical analysis indicated a weak association of tissue-DMRs with cultivars and growth conditions (*X*^2^ = 1885, *p* < 0.001; Cramer’s V = 0.108, 95% CI [0.103, 0.113]). Notably, only approximately 22% of the tissue-DMRs under stress overlapped with those from the control condition. The overlapped DMRs were predominantly in the CHH context. Most of the genes associated with DMRs were distinct from those detected under control conditions (**Figure 4A**). Under stress, the preference for hyper- or hypo-methylation of tissue-DMRs continued to show strong association with sequence contexts in N22 (*X*^2 =^ 7272, *p* < 0.001; Cramer’s V = 0.51, 95% CI [0.49, 0.52]). In contrast, the association was weaker in IR64 (*X*^2 =^ 148, *p* < 0.001; Cramer’s V = 0.06, 95% CI [0.04, 0.07]). Approximately 86% of total CHH tissue-DMRs in N22 were preferentially hypo-methylated, and 53% of CHG were hyper-methylated under stress (**Figure 2A**, right panel).

In the panicle, 57,237 cultivar-DMRs were identified under stress, representing an 18% decrease compared to the control condition. Across both cultivars, there were higher proportions of CHG (42%; 23,952) and CHH (34%; 19,519). However, unlike in the control, the association of hyper- or hypo-cDMRs with the sequence context was weak under stress (Cramer’s V = 0.21, 95% CI [0.20, 0.22]; *X*^2 =^ 2481, *p* < 0.001). This indicated an increase in hyper-methylated cDMRs (in N22 compared to IR64) of CHH in the tissue, which were preferentially hypo-methylated under control conditions (**Figure 2B**). The gene elements were more closely examined to investigate the differences in the distribution pattern of cDMRs under control and drought stress conditions. For CHG, hypo-methylated cDMRs reduced across the gene regions, while hyper-methylated cDMRs increased for CHH under stress (**Figure 4B**; **Supplementary Figure S7A**). In the flag leaf, 23,458 cDMRs were identified, with a notable increase under drought stress. A marginal rise in hypo-methylation of cDMRs was also observed for both CHG and CHH contexts under stress (**Figure 2C**). The distribution of cDMRs in the gene regions remained largely comparable to that observed under control conditions (**Supplementary Figure S7B**).

Both tissues may exhibit a similar pattern of cDMRs under control and stress conditions, but the overlapped cDMRs between the growth conditions were <29%. Of the total genes with cDMRs under stress, 30% (6,534) genes in the panicle and 60% (6,989) in the flag leaf were distinct compared to the control (**Figure 4C**). This underscores the distinctness in the methylation dynamics between control and drought stress.

### Drought-induced differentially methylated regions

**Figure 5:**
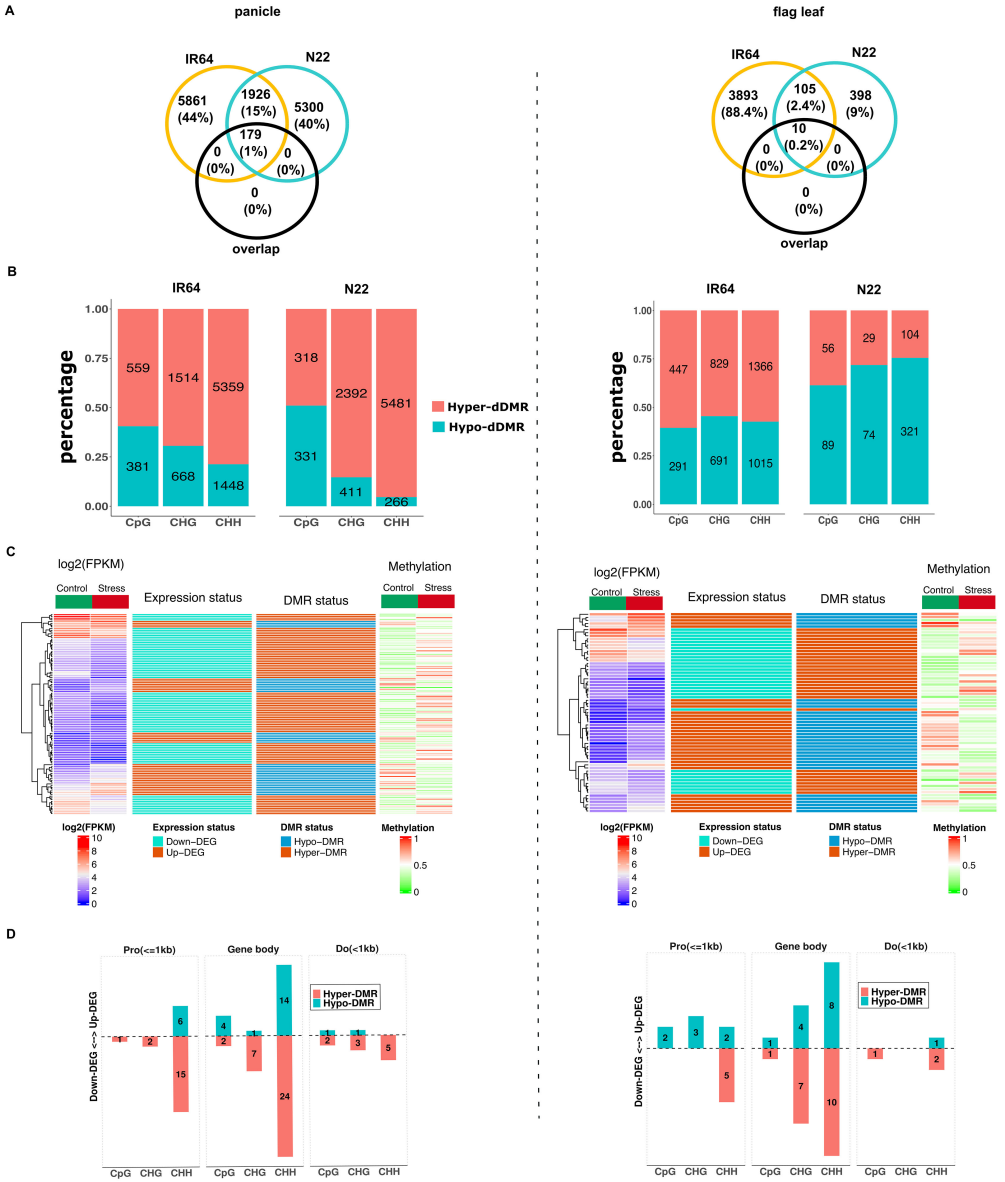
Drought-induced differentially methylated regions of panicle (right panel) and flag leaf (left panel). **(A)** Overlapped genes between IR64 (yellow circle) and N22 (cyan circle) that are associated with drought-induced DMRs. “Overlap” (black circle) represents the number of genes with overlapped dDMRs between the two cultivars. **(B)** Share of hyper- and hypo-dDMRs in N22 and IR64. Numeric value in the bar represents the number of dDMRs. **(C)** Heatmap of expression of DEGs that are negatively correlated with the methylation level of dDMRs under drought stress in IR64, shown only for IR64. Log2(FPKM) and methylation represent the average expression and methylation level of the genes under control and stress. Expression status represents whether the genes are upregulated or downregulated under stress. Likewise, DMR status represents whether hyper- or hypo-methylated in the genes under stress. **(D)** Position of dDMRs that are negatively correlated with expression of DEGs, shown only for gene regions within 1 kb upstream and downstream of genes in IR64. Number in the bar represents the number of DEGs. DMRs, differentially methylated regions; DEGs, differentially expressed genes.

To analyze the differences in methylome between control and drought stress within cultivar, DMRs within the 3-kb upstream and 3-kb downstream regions of the gene were again determined. These DMRs are referred to as drought-induced DMRs (dDMRs). In the panicle, a total of 9,929 dDMRs were detected in IR64 and 9,199 dDMRs in N22 (**Supplementary Table S9**). More than 60% of the dDMRs occurred in CHH contexts in both the cultivars, indicating strong sequence context preference (IR64: Cramer’s V = 0.763; *X*^2^ = 5776.5, *p* < 0.001; N22: Cramer’s V = 0.681; *X*^2^ = 4271.8, *p* < 0.001). The dDMRs of both the cultivars were enriched in gene body (30% in IR64 and 28% in N22), distal intergenic regions (21% in IR64 and 23% in N22), and promoter within 1-kb regions (16% in both). The comparative analysis of dDMRs between cultivars revealed only 234 overlapping regions in the panicle, representing less than 3% of the total, suggesting that most dDMRs are cultivar-specific. More importantly, the majority of genes associated with dDMRs differed between the two cultivars (**Figure 5A**, left panel).

In both cultivars, panicles showed a higher number of hyper-methylated dDMRs (**Figure 5B**, left panel). However, N22 had more hypo-methylated dDMRs than hyper-methylated ones in the CpG context, unlike IR64. Statistical analysis also indicated a stronger association between dDMRs status and sequence contexts in N22 compared to IR64 (N22: Cramer’s V = 0.38, 95% CI [0.35, 0.41]; *X*^2^ = 1342, *p* < 0.001; IR64: V = 0.14, 95% CI [0.12, 0.17], *X*^2 =^ 207, *p* < 0.001). This suggests that in N22, dDMRs of CHH and CHG contexts were preferentially hyper-methylated compared to CpG, but no such preferences were observed in IR64.

Drought-induced DMRs in the flag leaf were estimated to be 4,369 and 673 in IR64 and N22, respectively (**Supplementary Table S9**). The majority of these dDMRs constituted CHH contexts (51% in IR64 and 63% in N22). In IR64, CHG accounted for 33% of the total dDMRs, while CpG constituted the smallest proportion (16%). Conversely, N22 had a higher proportion of CpG (22%) compared to CHG (15%). The context preference for dDMRs in the flag leaf was practically significant (IR64: Cramer’s V = 0.44, *X*^2 =^ 873.53, *p* < 0.001; N22: Cramer’s V = 0.637, *X*^2 =^ 273.18, *p* < 0.001). Similar to panicle, dDMRs were enriched in the gene body and intergenic regions. The overlap of dDMRs between the two cultivars was less than 3% (14), with many distinct genes identified between the cultivars (**Figure 5A**, right panel). In IR64, 57% of dDMRs were hyper-methylated, compared to only 28% in N22 (**Figure 5B**, right panel). There was weak context preference for hyper- or hypo-methylation in the flag leaf (IR64: Cramer’s V = 0.0, *X*^2 =^ 7.7, *p* = 0.021; N22: 0.11, *X*^2 =^ 10.7, *p* = 0.005).

### Drought-induced DMRs and gene expression

In the panicle, there were a total of 115 differentially expressed genes (DEGs) in IR64 and 293 in N22 that showed a negative correlation with the methylation status of dDMRs (**Supplementary Table S10**). In the flag leaf, 65 DEGs were identified in IR64 and 10 DEGs in N22 with negative correlations. More DEGs had expression negatively correlated with hyper-methylated dDMRs in the panicle, but the opposite was observed in the flag leaf (**Figure 5C**; **Supplementary Figure S8**). Furthermore, the expression of DEGs associated with dDMRs of CHH context exhibited a stronger negative correlation with methylation pattern than other sequence contexts (**Figure 5D**; **Supplementary Figure S9**). Ontology analysis of the downregulated DEGs under drought stress associated with hyper-methylated dDMRs revealed significant enrichment in biological processes related to actin filament regulation, negative regulation of actin filament depolymerization, and cytoskeleton organization in the panicle. Notably, OsTOP6A1 (*Os03g54091*), previously reported to confer dehydration tolerance (Jain et al., 2008), was downregulated in IR64 in association with CHH hyper-methylation (dDMRs) within its gene body. Auxin-responsive genes (OsGH3-3; *Os01g12160*), with hyper-methylated CHH in the promoter within 1-kb regions, were also downregulated. Additional downregulated genes included OsMADS27 (*Os02g36924*), bZIP transcription factor domain-containing protein (*Os01g11350*), and a gene with a putative function of HEAT repeat family protein (*Os11g04220*) in the panicle. In contrast, most downregulated DEGs that co-localized with hyper-dDMRs in N22 were related to membrane transportation and metabolic processes. WRKY69 (*Os08g29660*) and some genes considered as MYB family transcription factor (*Os04g42950*, *Os05g37730*, *Os07g43580*, and *Os08g22800*) were also downregulated in the cultivar.

Genes upregulated under stress and associated with hypo-dDMRs in the panicle of N22 include those involved in oxidative stress response (*Os12g08830*), MYB transcription factors (*Os05g10690*, *Os02g36890*, *Os02g36890*, and *Os0645890*), late embryogenesis abundant protein (*Os01g50910*), and stress-related protein (*Os05g05940*). The auxin-responsive genes (OsSAUR55; *Os09g37500*) were also upregulated in N22. Additionally, genes implicated in grain filling, such as OsSCP26 (*Os05g06660*) and OsSCP46 (*Os10g01134*), showed increased expression. In IR64, upregulated genes were primarily associated with metabolic processes, including two stress-responsive genes (*Os03g51330* and *Os09g28354*) and three putative retrotransposon genes (*Os02g26500*, *Os09g07940*, and *Os07g23640*) in the panicle. In the flag leaf tissue of IR64, oxidative stress-related genes (*Os12g18360* and *Os10g38700*) were downregulated, while a dehydration-responsive gene (*Os10g36690*) was upregulated in association with hypo-dDMRs.

The result suggests that in the tolerant cultivar, hypo-methylation facilitates the activation of key adaptive genes, indicating a role of DNA methylation as a positive regulator of drought tolerance. Conversely, in the drought-sensitive IR64, drought triggers hyper-methylation-mediated repression of adaptive genes.

### Drought-specific cultivar-DMRs and differentially expressed genes

In the panicle, 7,191 and 5,076 genes (both DEGs and non-DEGs) were associated with drought-specific hyper- and hypo-methylated cDMRs, respectively (**Supplementary Figure S10B**). The drought-specific cDMRs were enriched in the exon and promoter within 1-kb regions (**Supplementary Figure S10D**). Across gene elements, CpG and CHG exhibited a higher frequency of hyper-methylation, whereas CHH methylation showed a relatively balanced distribution of hyper- and hypo-cDMRs. A total of 1,353 Differentially Expressed Genes between the cultivar (cDEGs) (787 downregulated and 566 upregulated) displayed expression levels that were negatively correlated with the methylation status of drought-specific cDMRs. Among these, the cDMRs of CHH were associated with 570 negatively correlated cDEGs, while CpG and CHG corresponded to 432 and 480 cDEGs, respectively. Ontology analysis showed that genes upregulated in N22 compared to IR64 were involved in stress responses such as oxidative and heat stress (*Os01g49290*, *Os02g54140*, *Os03g14180*, *Os04g09900*, *Os04g48410*, *Os06g11280*, *Os07g48030*, *Os08g27070*, and *Os08g39840*), as well as flower development (*Os01g15340*, *Os02g26210*, and *Os10g35110*) and other developmental processes. In contrast, downregulated genes in N22 associated with hyper-cDMRs were mostly involved in metabolic and biosynthetic processes.

In the flag leaf, there were 5,834 drought-specific hypo-cDMRs and 2,406 hyper-cDMRs (**Supplementary Figure S11B**). Among these, the expression of 66 upregulated and 117 downregulated cDEGs showed a negative correlation with the methylation status of drought-specific cDMRs (**Supplementary Figure S11C**). Similar to those in the panicle, a higher proportion of cDEGs (75) negatively correlated with CHH methylation compared to CpG (63) and CHG (60). Some of the downregulated cDEGs of N22 were hydrolases, involved in glucosidase activity and other metabolic processes, whereas upregulated cDEGs with drought-specific hypo-cDMRs were involved in membrane transportation activities and metabolic processes.

In the tolerant cultivar, hypo-methylation of stress-responsive genes facilitates their activation, which may enhance stress responses and promote adaptive developmental processes like flowering under drought. In contrast, hyper-methylation of metabolic genes may help conserve energy by downregulating non-essential biosynthetic pathways during stress.

### MiRNome methylation profile in N22

We had previously generated small RNA data for the heading and anthesis stages of N22 under control and field drought conditions (Kansal et al., 2015; Balyan et al., 2017). Hence, we sought to find the methylation status of the miRNA genes in the N22 genome and learn whether it affects the miRNA expression or not. We used the different structural regions of a miRNA gene [mature miRNA, precursor, promoter taken as upstream of precursor start site (0–1, 1–2, and 2–3 kb), and downstream region taken as downstream of precursor end site (0–1, 1–2, and 2–3 kb)] as features for finding differentially methylated regions. As a result, we found several differentially methylated features (DMFs) under drought stress in both tissues. Similar to protein-coding genes, drought stress, interestingly, induced more hyper-methylated DMFs in the panicle while more hypo-methylated DMFs in the flag leaf (FL). However, in both tissues, most of the DMFs belong to the CHH context and lie within promoter and downstream regions.

**Figure 6:**
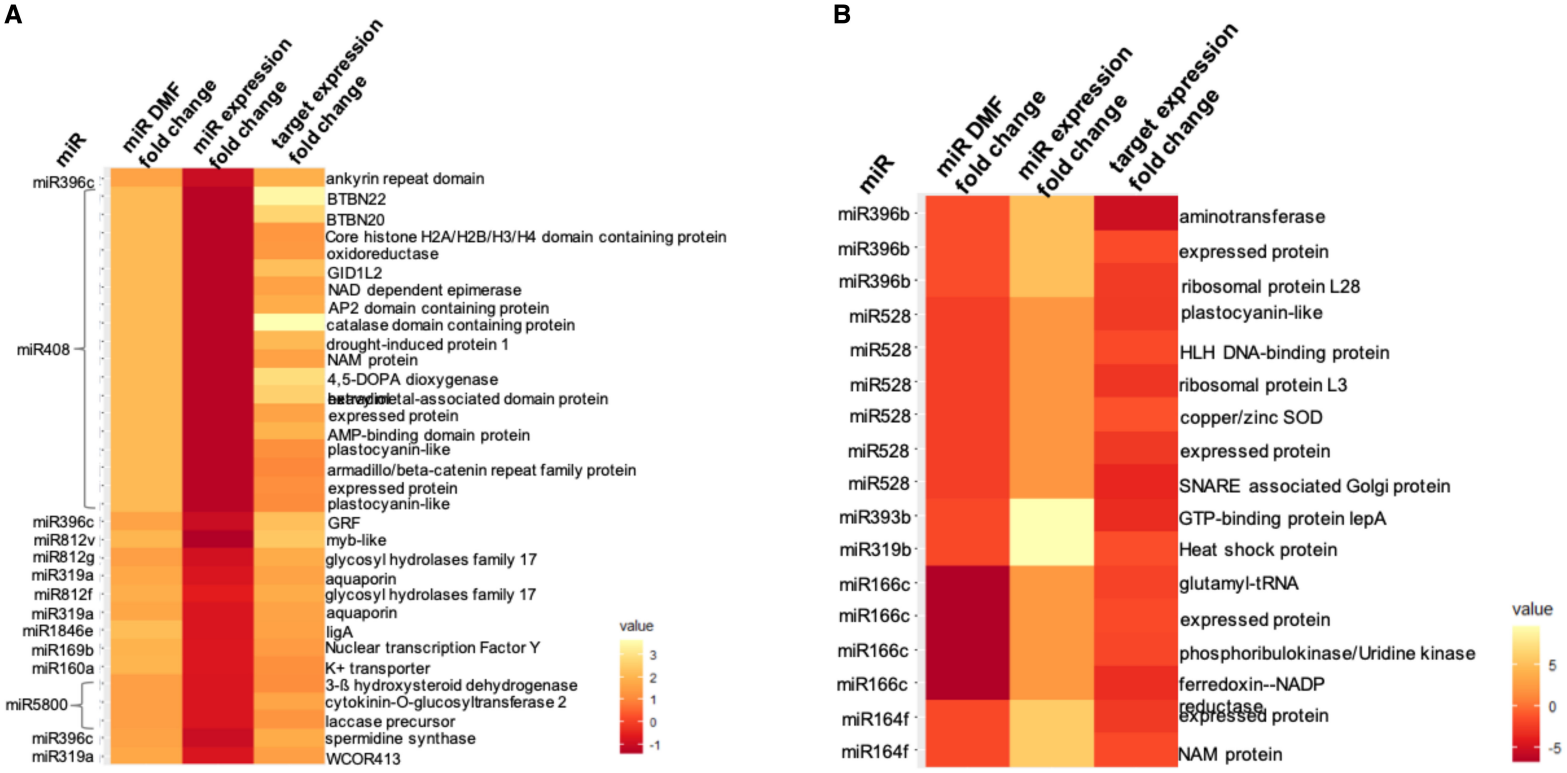
Heatmap representation of differentially methylated features (DMFs) associated with miRNA genes under drought stress **(A)** in N22 panicle and **(B)** between panicle and flag leaf. The associated change in expression of the miRNA and its targets can also be seen. The fold changes are log2[fold change (stress/control)].

Studying the DMFs found in the panicle, some miRNAs showed interesting methylation profiles with more than one feature showing differential methylation in similar or different contexts. For instance, miR319a had its precursor (in the CHH context) as well as promoter 0–1 kb (in the CpG context) hyper-methylated under drought, while the expression of the mature miR319a decreased. Similarly, miR812g also showed a decline in expression along with hyper-methylated downstream regions (0–1 kb in the CpG context and 2–3 kb in the CHH context). MiR812f had its promoter 2–3 kb and downstream 2–3 kb hyper-methylated in the CHH context, and the expression of its mature form decreased under drought. Interestingly, more than half of the DMFs that showed anti-correlation with the miRNA expression fell in the downstream region, thereby highlighting the significance of the region downstream of the precursor. This region may be part of a primary-MIRNA transcript and may have regulatory roles in its processing/biogenesis. We further analyzed the targets of the miRNAs, showing an anti-correlation between their methylation status and expression levels. In N22 panicle, most of the DMFs were hyper-methylated in different contexts along with a decline in the miRNA expression and subsequent significant upregulation of their target genes (DEGs under drought; Gour et al., 2022) (**Figure 6A**). In this analysis, miR408 was noticeable, which had a hyper-methylated precursor (CHG context) and showed a decline in its expression in response to drought. A large number of targets were predicted for miR408, and among them, many were significant DEGs, including plastocyanin-like protein, oxidoreductase, drought-induced protein 1, catalase domain-containing protein, and more in upregulated expression. Additionally, aquaporin and cold acclimation protein WCOR413 were found to be upregulated against a downregulated miR319a (hyper-methylated precursor and promoter); ankyrin repeat domain-containing protein, a growth-regulating factor TF, and spermidine synthase were upregulated due to a decline in miR396c expression (hyper-methylated downstream region). Multiple catalytic enzymes targeted by miR5800 were also upregulated under drought due to downregulation in the miRNA expression (hyper-methylated downstream region). Furthermore, two loci encoding for glycosyl hydrolase family 17 were enhanced under stress due to decreased miR812f and miR812g (hyper-methylated downstream or promoter region). Considering all the predicted targets, the Gene Ontology (GO) terms that became enriched included “transcription factor activity”, “oxidoreductase”, “water channel activity”, and “ferredoxin-NADP+ reductase activity”.

In the FL, no DMFs could be found for the CHG context, while only one could be found for the CpG context. CHH context showed 14 DMFs, most of which were hypo-methylated. Incidentally, most of the DMFs associated with miRNAs were either not detected in our data or not significantly differentially expressed under drought. One hyper-methylated DMF overlapped with miR162a downstream (2–3 kb) region, while the miRNA became highly repressed in FL drought. Similarly, miR169l had its promoter (2–3 kb) hypo-methylated, while the expression of the mature miRNA was upregulated in FL under stress. Furthermore, the predicted targets were found to be non-differential under drought in the transcriptome data.

Comparing the two tissues under non-stress control conditions gave us an idea about tissue-preference or tissue-specific expression profiles of miRNAs and their methylation states. Hence, we analyzed the DMFs between panicle and FL under control conditions. Here, more than half of the DMFs found belonged to the CpG context relating to several miRNAs (**Figure 6B**). For instance, two different members of the miR812 family showed hypo-methylation at more than one feature—miR812d showed hypo-methylation at mature, precursor, and downstream 0–1-kb regions, while miR812e was hypo-methylated at promoter 2–3 kb as well as downstream 0–1 kb, all in the CpG context. Since both members produced identical mature miRNAs, the expression profile showed a greater expression of the mature miRNA form in the panicle than in FL. Similarly, miR818c showed hypo-methylation at promoter 2–3 kb (CHH), while miR818d showed hypo-methylation at promoter 0–1 kb along with downstream 0–1 kb (CpG). The mature form of both members was identical and showed multi-fold greater presence in the panicle than FL. Additionally, a case of the miR171 family, where miR171b showed a hypo-methylated promoter 2–3 kb (CpG) while miR171f showed hypo-methylated downstream 1–2 kb (CHH), was also present. Both members produced distinct forms of mature sequence, and both of these mature forms became upregulated in the panicle vis-à-vis FL. In our miRNome data, we showed some panicle-specific miRNAs that had a significant presence in one tissue (TPM > 5) while being absent in the other tissue. On the same lines, miR5806 is a panicle-specific miRNA that showed hypo-methylation at promoter 2–3 kb as well as downstream 1–2 kb. Several other miRNAs also showed greater expression in the panicle as compared to FL and have hypo-methylated features. Such examples include miR528, miR5498, miR319b, miR166c, miR393b, miR164f, miR1861m, and miR396b (**Figure 6B**). The targets of some of these aforementioned miRNAs showed anti-correlation with the miRNAs, as shown in **Figure 6B** prominently. Several targets predicted for miR528 showed anti-correlation, such as plastocyanin-like protein, HLH DNA-binding protein, Cu/Zn SOD, and SNARE-associated Golgi protein. MiR166c was predicted to target glutamyl-tRNA, phosphoribulokinase/uridine kinase, ferredoxin NADP reductase, and an unannotated protein-encoding gene, which were all downregulated in the panicle as compared to the FL.

## Discussion

The regulation of DNA methylation signature is critical for plant development (He et al., 2022; Liang et al., 2022; Williams et al., 2022) as well as in the regulation of drought stress responses (Li et al., 2020; Wang et al., 2021; Van Dooren et al., 2020; González et al., 2013; Wang et al., 2011; Duan et al., 2020). In the rice seedlings of N22 (tolerant) and IR64 (sensitive) cultivars, drought-induced change in DNA methylation was reported to play regulatory roles in the expression of genes responsible for stress tolerance (Rajkumar et al., 2020; Garg et al., 2015). Likewise, an examination of the DNA methylation patterns of DK151 (tolerant) and IR64, cultivated in a greenhouse, highlighted the variation and impact of methylation on gene expression related to drought stress (Wang et al., 2016, Wang et al., 2011). These studies indicate the significance of methylation in drought-tolerant and drought-sensitive rice cultivars. The present study emphasized how methylation response varied between the two contrasting rice genotypes at the mature stage (“heading”) in a field environment under control and drought stress.

### IR64 and N22 maintain an inherent unique global methylation signature under both control and drought conditions

The pattern of cytosine composition, with CpG being the most prevalent, is consistent with observations in flowering plants (Kou et al., 2021; Rajkumar et al., 2020; Song et al., 2013; Lister et al., 2008; Feng et al., 2010). In our study, we observed many overlapping cytosines between the two contrasting cultivars with marginal differences in the methylation level, regardless of the growth conditions and tissues. This suggests the stability and conserved nature of the methylome, aligning with the findings that indicated high conservation of methylation in rice, irrespective of genotype variations (Wang et al., 2016). Notably, this propensity for methylation stability has also been reported in *Arabidopsis* (Ganguly et al., 2017; Becker et al., 2011). However, even though the global pattern appeared similar under control conditions, a more in-depth examination revealed differences in the variations among the cytosine contexts between cultivars. We noticed that there was greater variation in the CHH context when compared to CpG and CHG between cultivars. A comparison between the shoot apical meristem and mature leaf in rice also indicated that CHH displayed more pronounced dynamics compared to other contexts (Higo et al., 2020). The authors proposed that excessive methylation of CHH is necessary to effectively suppress transposable elements. These finding underscores the intricate interplay of methylation dynamics in different sequence contexts, which seem to be inherent even in the absence of drought stress. This intriguing observation may also signify the outcome of multiple generations of adaptation to stress conditions, which increases the potential for adaptation (Hauben et al., 2009; Verhoeven et al., 2010; Molinier et al., 2006). Moreover, in our study, we observed that this heightened variability in CHH was consistent across the two tissues under stress. Notably, several of the drought-induced or drought-specific methylation variations and genes that associate with such DMRs were distinct between the growth conditions and cultivars.

In brief, while the whole-genome methylated cytosines may exhibit a high level of conservation, the sequence preferences for hypo- and hyper-methylation, particularly in the CHH context, and their association with genes vary significantly between the cultivars. Such methylation variation signatures differ between control and drought stress conditions.

### Significance of drought-induced CHH methylation dynamics

DNA methylation plays a prominent role in modulating the chromatin structure (Hashimshony et al., 2003). It introduces local modifications to the structural characteristics of DNA, which in turn can lead to alterations of DNA–protein interactions (Kribelbauer et al., 2020; Domcke et al., 2015; Ko et al., 2005). A notable observation in our study is the prevalence of DEGs with expression negatively correlated with hyper-methylated dDMRs in the panicle, suggesting a potential preference for hyper-methylation as a response mechanism to drought stress in the tissue. However, hypo-methylation was more prevalent in the flag leaf. Moreover, when considering sequence contexts, DEGs associated with dDMRs of the CHH sequence context exhibited stronger negative correlations with methylation patterns compared to other sequence contexts. More than 50% of the dDMRs associated with DEGs were of CHH contexts. In plants, the impact of methylated sequence contexts on DNA–protein interactions can vary. For instance, in rice endosperm, the methylation status of CpG and CHG sequences has been identified as having a significant influence on gene transcription (Zemach et al., 2010). Similarly, in *Arabidopsis*, CpG methylation is recognized to exert a more pronounced effect on gene expression compared to non-CpG methylation (He et al, 2022). However, the significance of the CHH sequence context appears to become more prominent under stress conditions. In *Arabidopsis*, methylation of the CHH sequence context has been highlighted for its critical role in responding to biotic stress (Le et al., 2014). Moreover, in the context of drought stress, CHH methylation has been found to exhibit a stronger correlation with gene expression changes induced by drought compared to other sequence contexts in rice seedlings (Rajkumar et al., 2020). The sequence context, in general, appears to have a particular significance for the normal functioning of the genome in rice (Higo et al., 2020; Kou et al., 2021; Secco et al., 2015; Wang et al., 2021). The presence of the CHH island in maize also holds significance in terms of coordinating cellular gene expression alongside the concurrent repression of nearby transposons (Gent et al., 2013).

A deeper examination of the DEGs associated with dDMRs and cDMRs highlights the functional relevance under drought stress. Some of the drought-responsive genes and auxin-responsive genes were downregulated in association with dDMRs of CHH in IR64. The downregulated OsTOP6A1 (*LOC_Os03g54091*) gene of IR64 is known to induce dehydration tolerance when constitutively expressed in *Arabidopsis* (Jain et al., 2008). MADS genes (*LOC_Os02g36924* and *LOC_Os05g11414*), which are considered to be important floral homeotic genes involved in specifying rice flower development (Dreni et al., 2011), were also downregulated in IR64 in association with dDMRs of CHH. In contrast, N22 exhibited drought-induced hypo-methylation of cDMRs in flower-related genes (*LOC_Os01g15340*, *LOC_Os02g26210*, and *LOC_Os10g35110*), accompanied by their upregulation under stress relative to IR64. This contrasting pattern suggests that cultivar-specific methylation changes modulate the expression of flowering regulation genes in opposite directions under drought conditions. Furthermore, N22 showed enhanced expression of hypo-methylated genes involved in grain filling, auxin response, oxidative stress response, and heat stress under drought stress.

These findings highlight the distinct methylation dynamics between the two cultivars. In N22, hypo-methylation is associated with transcriptional activation of key adaptive pathways, supporting its drought tolerance, whereas in IR64, hyper-methylation correlates with repression of stress-responsive genes, reflecting a negative regulatory role of methylation in stress adaptation.

### DNA methylation impact on drought-responsive miRNAs

There have been many previous reports identifying differential methylation of miRNA loci in rice and other plant species (Lu et al., 2016; Song et al., 2015; Ci et al., 2015; Ganie et al., 2016; Song et al., 2016; Liu et al., 2017; Hua et al., 2020; Piya et al., 2021). The transcription of the miRNA genomic locus is dependent on the region upstream of the precursor, often considered as its promoter. Thus, differential methylation of promoter and precursor may lead to differential binding of the transcription machinery and subsequently its expression. In our study, we found DMFs in many known drought-responsive miRNAs, such as members of miR166, miR156, miR812, miR408, and miR319. MiR408 was noticeable, which showed anti-correlation in expression with many of its targets, including plastocyanin-like and more catalytic enzymes. Differential methylation may be a way to regulate the expression of miRNAs under drought stress, and the result was often fine-tuning of the target genes. Similarly, miR528, miR396b, miR166c, and miR164f were the miRNAs that showed anti-correlation in expression with many of their predicted targets in the inter-tissue comparison (panicle *vs*. flag leaf). The end result was downregulation of many structural (ribosomal proteins and SNARE-associated Golgi protein) and catalytic proteins (Cu/Zn SOD, plastocyanin-like, and uridine kinase) in the panicle compared to the flag leaf. The presence of a significant number of DMFs in the downstream region indicated an undiscovered role of this region in regulating the expression of the mature miRNA. Interestingly, for some miRNAs, the precursor along with promoter regions or promoter along with downstream regions were similarly hyper- or hypo-methylated in the same or different contexts. Such heavy differential methylation between conditions or tissues highlights the prominent role of DNA methylation in the regulation of genomic loci.

In summary, the study revealed that while genome-wide DNA methylation patterns in rice remain largely conserved under both control and drought conditions, notable cultivar-specific changes emerge in response to drought, particularly within the CHH context. These methylation reprogramming events suggest an active epigenetic component potentially contributing to the distinct drought tolerance observed between the two contrasting cultivars. Furthermore, differences in the association of methylation with key metabolic, developmental, and stress-responsive pathways underscore the diverse regulatory strategies employed by IR64 and N22 in adapting to drought stress.

## Data Availability

The bisulfite sequencing data have been submitted to IBDC (study accession INRP000047) and the GenBank databases under accession number PRJEB58713.
