# Supplementary material for: Genome-wide DNA methylation dynamics at “heading” stage of panicle and flag leaf in contrasting rice cultivars under field drought conditions
Source: Front Plant Sci. 2025 Nov 11;16:1707950. doi: 10.3389/fpls.2025.1707950 (PMC12659711; doi:10.3389/fpls.2025.1707950)
Supplement: Supplementary file 1 [file DataSheet1.docx]

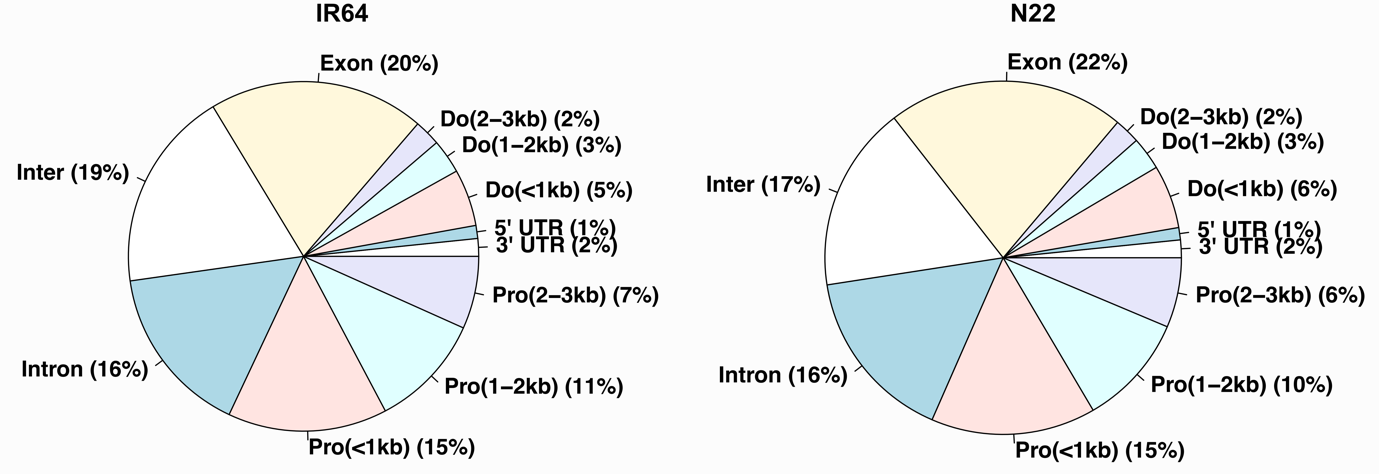


Figure S 1. Pie chart showing the distribution of methylated cytosine across gene elements of flag leaf in IR64 and N22. Pro – promoter, Do – downstream and Inter – Distal intergenic.


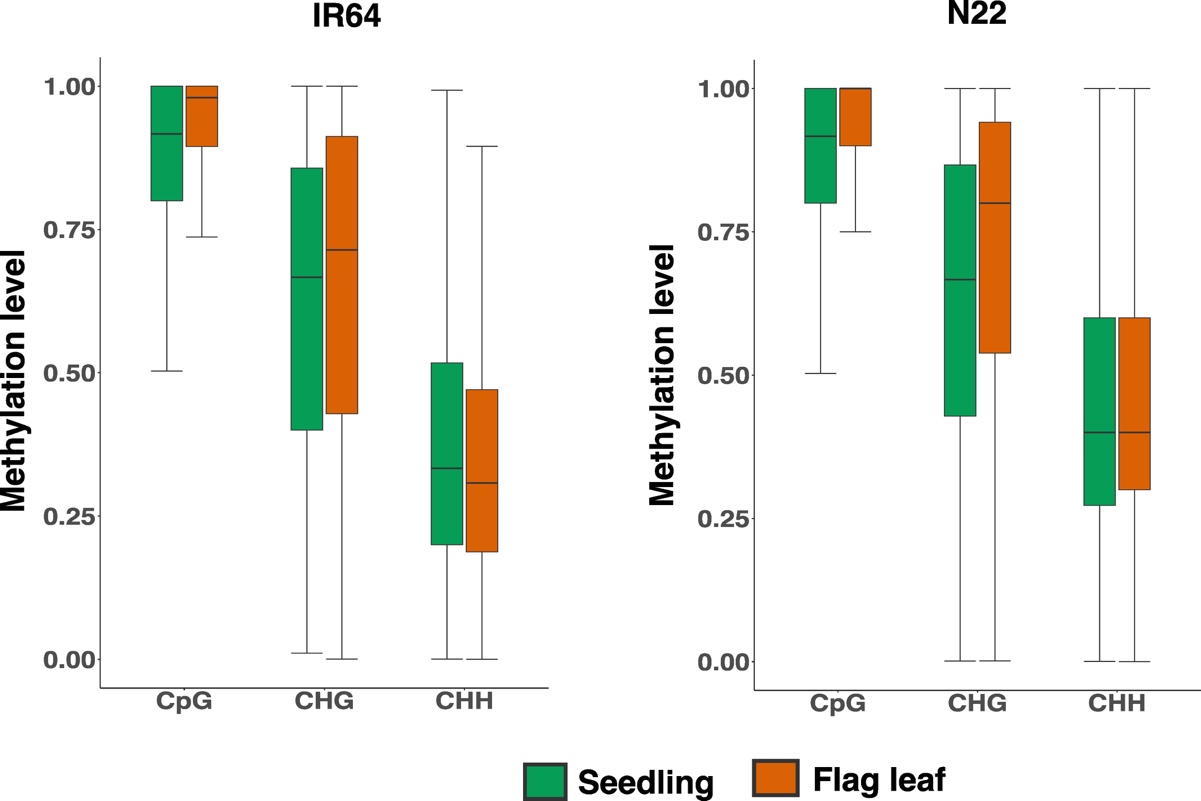


Figure S 2. Distribution of methylation level in flag leaf (mature tissue) and seedling. The seedling data is from a previous study (Garg et al., 2015).


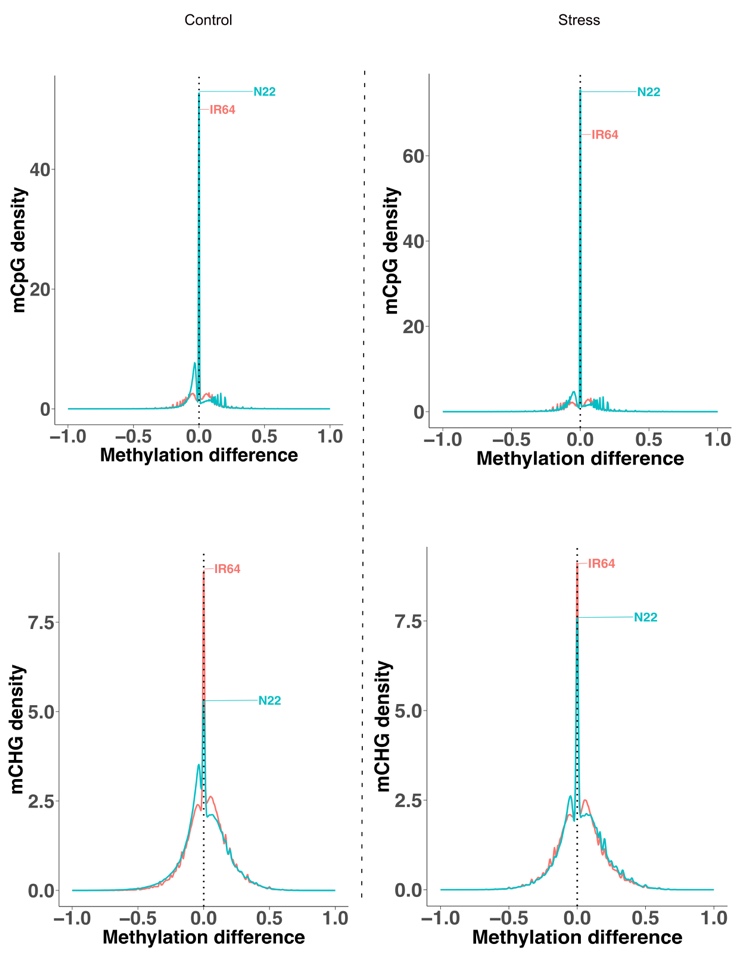


Figure S 3. Kernel density plot of methylation differences between panicle vs flag leaf under control and drought stress. Greater than zero indicates hyper-methylation in panicle compared to flag leaf and vice versa. Vertical dotted line represent no differences in methylation level between the tissues.


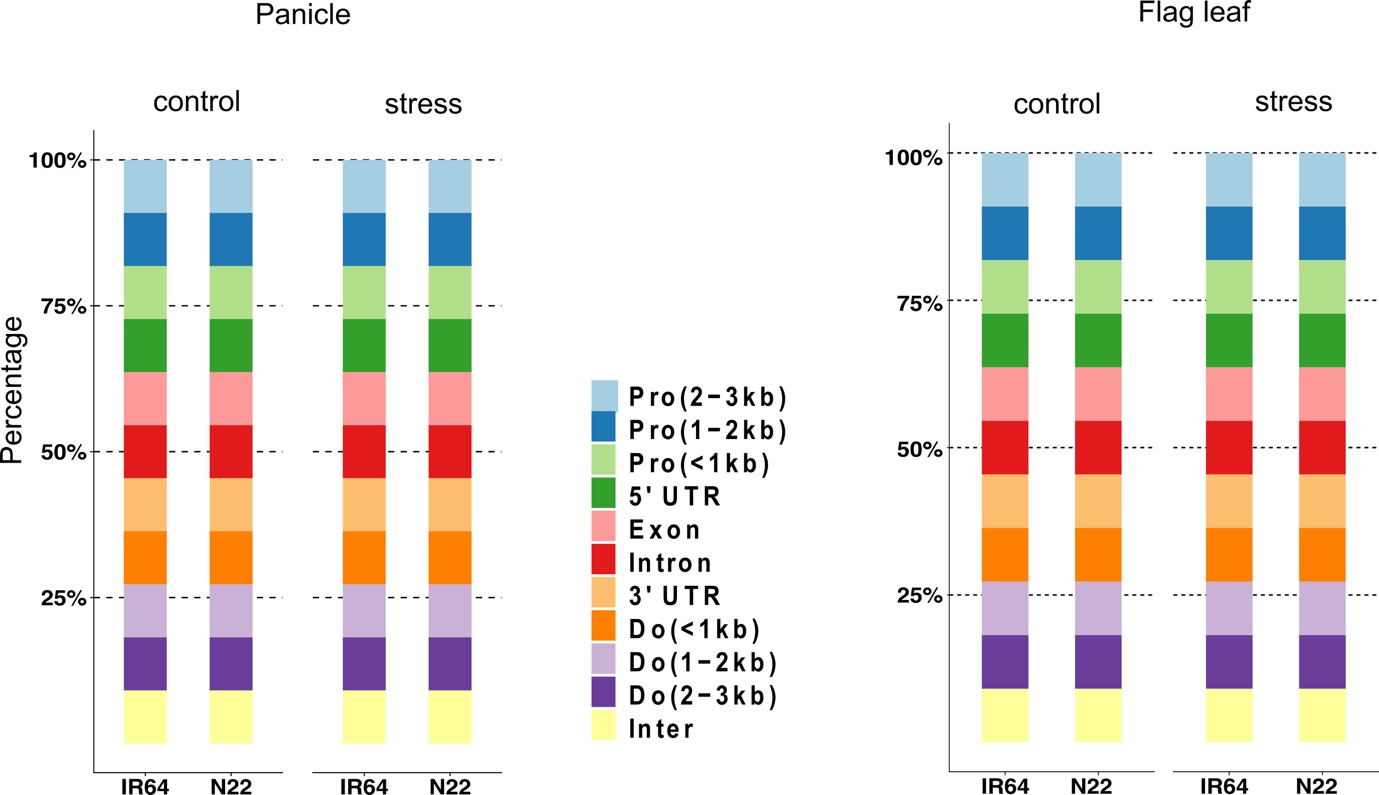


Figure S 4.Distribution of methylated cytosines across different gene elements under control and drought stress in the panicle and flag leaf. Pro – promoter, Do – downstream and Inter – distal intergenic.


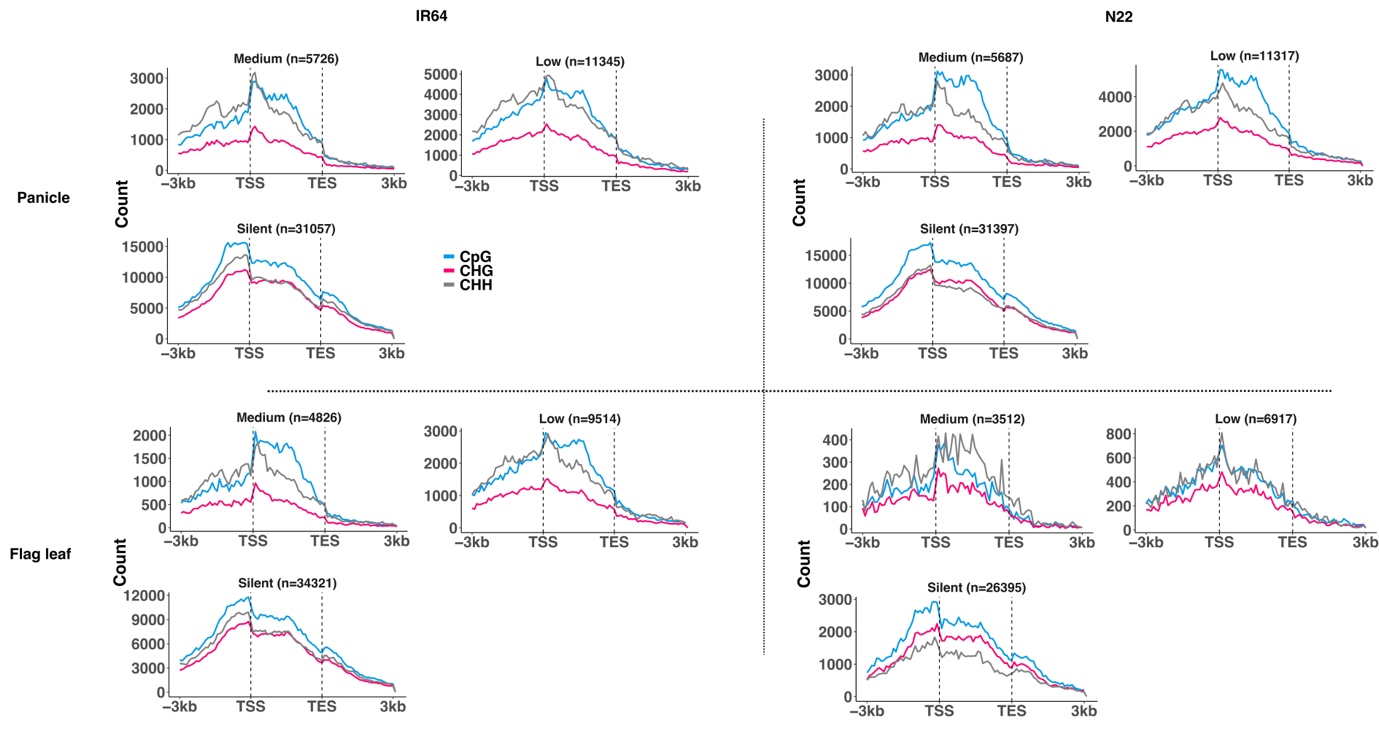


Figure S 5. Distribution of de-methylated cytosines under drought stress across the gene regions of panicle (upper panel) and flag leaf (lower panel) in IR64 (left panel) and N22 (right panel). Gene body was converted to proportion. Dotted vertical lines represent the alignment of transcription start site (TSS) and transcription end site (TES). “n” within the bracket indicates the number of genes for the expression category. Silent – gene with FPKM < 0.05, Low –genes within 2^nd^ quartile of expression and medium –genes within 3^rd^ quartile of expressions.


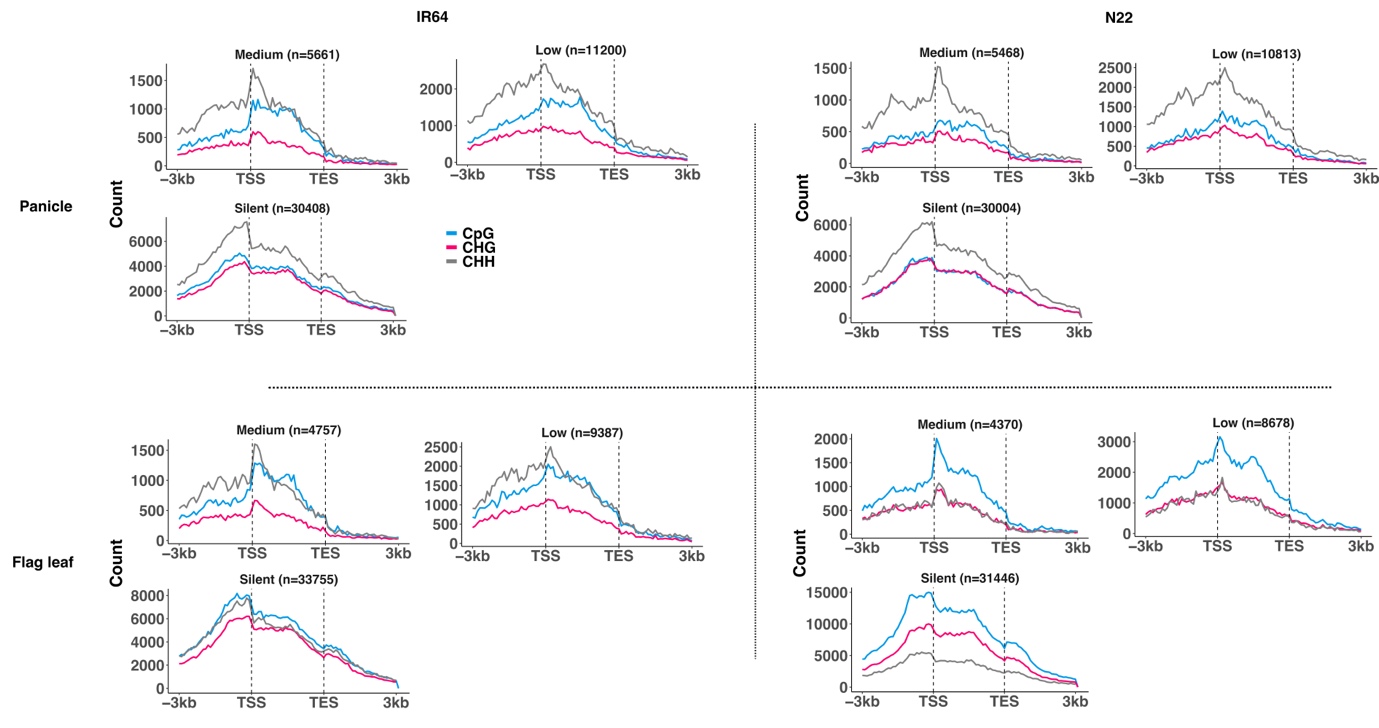


Figure S 6. Distribution of de novo methylated cytosines under drought stress across the gene regions of panicle (upper panel) and flag leaf (lower panel) in IR64 (left panel) and N22 (right panel). Gene body was converted to proportion. Dotted vertical lines represent the alignment of transcription start site (TSS) and transcription end site (TES). “n” within the bracket indicates the number of genes for the expression category. Silent – gene with FPKM < 0.05, Low –genes within 2^nd^ quartile of expression and medium –genes within 3^rd^ quartile of expressions.


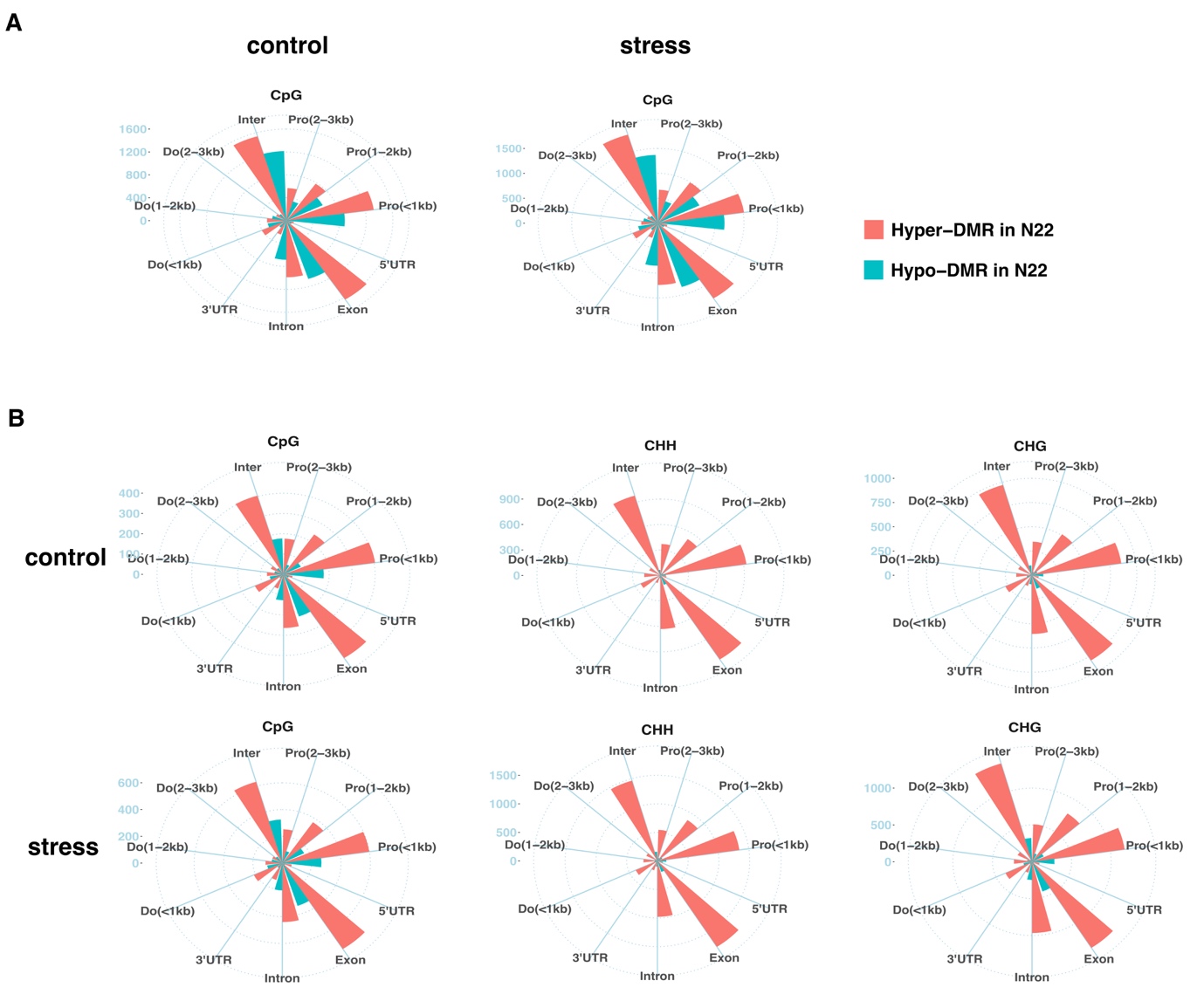


Figure S 7. Comparisons of distribution of hyper- and hypo-cDMR across different gene elements under control and drought stress. A) comparison of CpG distribution in panicle B) comparison of the three cytosine sequence contexts in flag leaf. Pro – promoter; Do – downstream and Inter – distal intergenic.


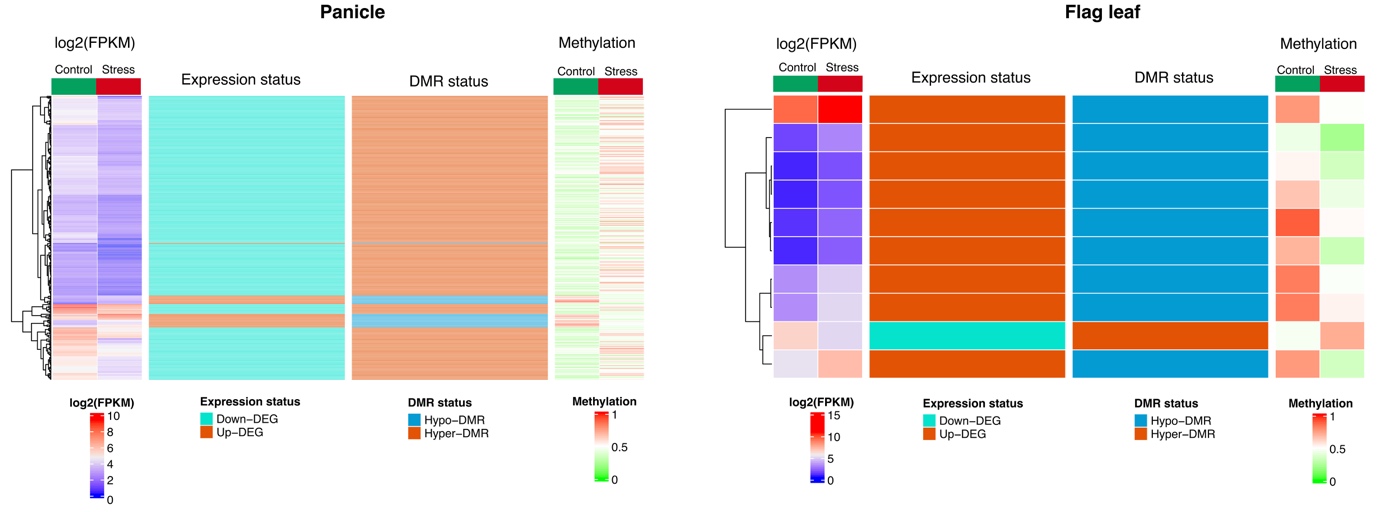


Figure S 8. Heatmap of expressions of DEG that are negatively correlated with the methylation level of dDMR under drought stress in N22. log2(FPKM) and methylation represents the average expression and methylation level of the genes under control and stress. Expression status represents whether the genes are up-regulated or down-regulated under drought stress. Likewise, DMR status represents whether hyper- or hypo-methylated in the genes under stress.


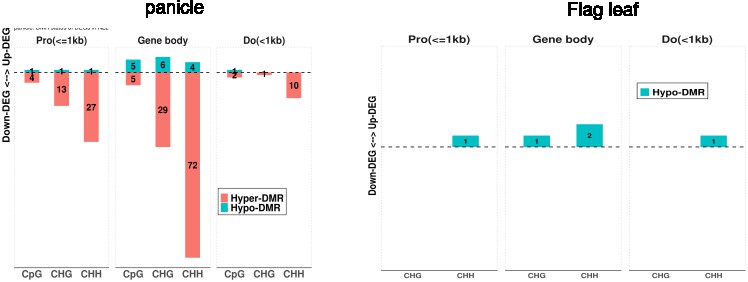


Figure S 9. Position of dDMRs that are negatively correlated with expressions of DEGs in N22. Shown only for gene region within 1kb upstream and downstream of genes. Number in the bar represents the number of DEG.


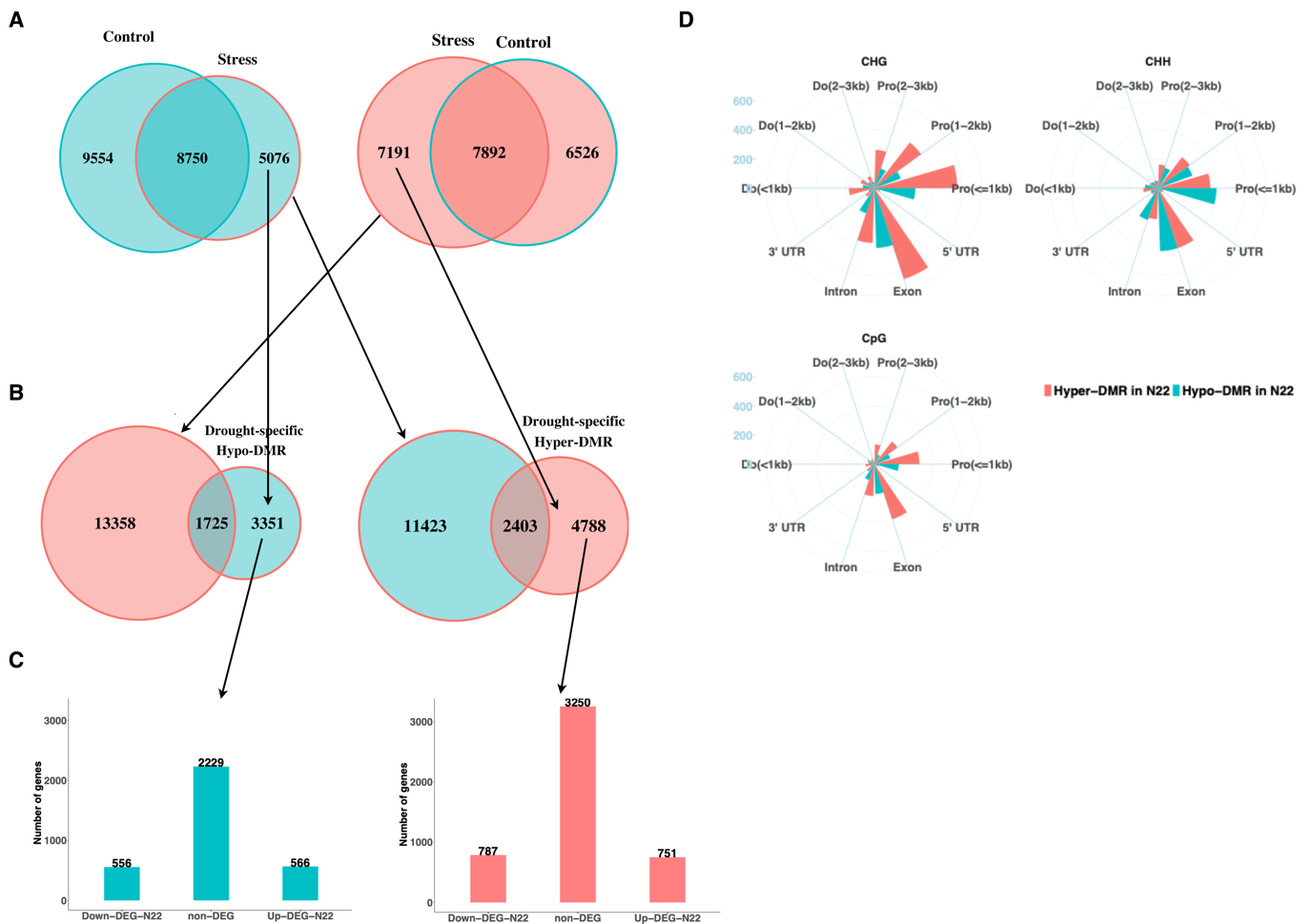


Figure S 10. Genes with drought-specific cDMR in panicle. A) total number of genes with hyper- and hypo-cDMR under control and drought stress. B) Genes with drought specific hyper- and hypo-cDMR. C) Expression status of genes with drought-specific cDMR. Down-DEG-N22 and up-DEG-N22 indicates down-regulated and up-regulated DEG in N22 compared to IR64, and non-DEG indicated non differentially expressed genes between the two cultivars. D) Distribution pattern of drought-specific hyper and hypo-cDMR across gene elements. Pro – promoter region, Do – Downstream region. Numeric value in the circle represents number of genes. Cyan and red color circumference of circles indicate control and stress. Blue and red color filled circles indicate hyper and hypo-cDMR.


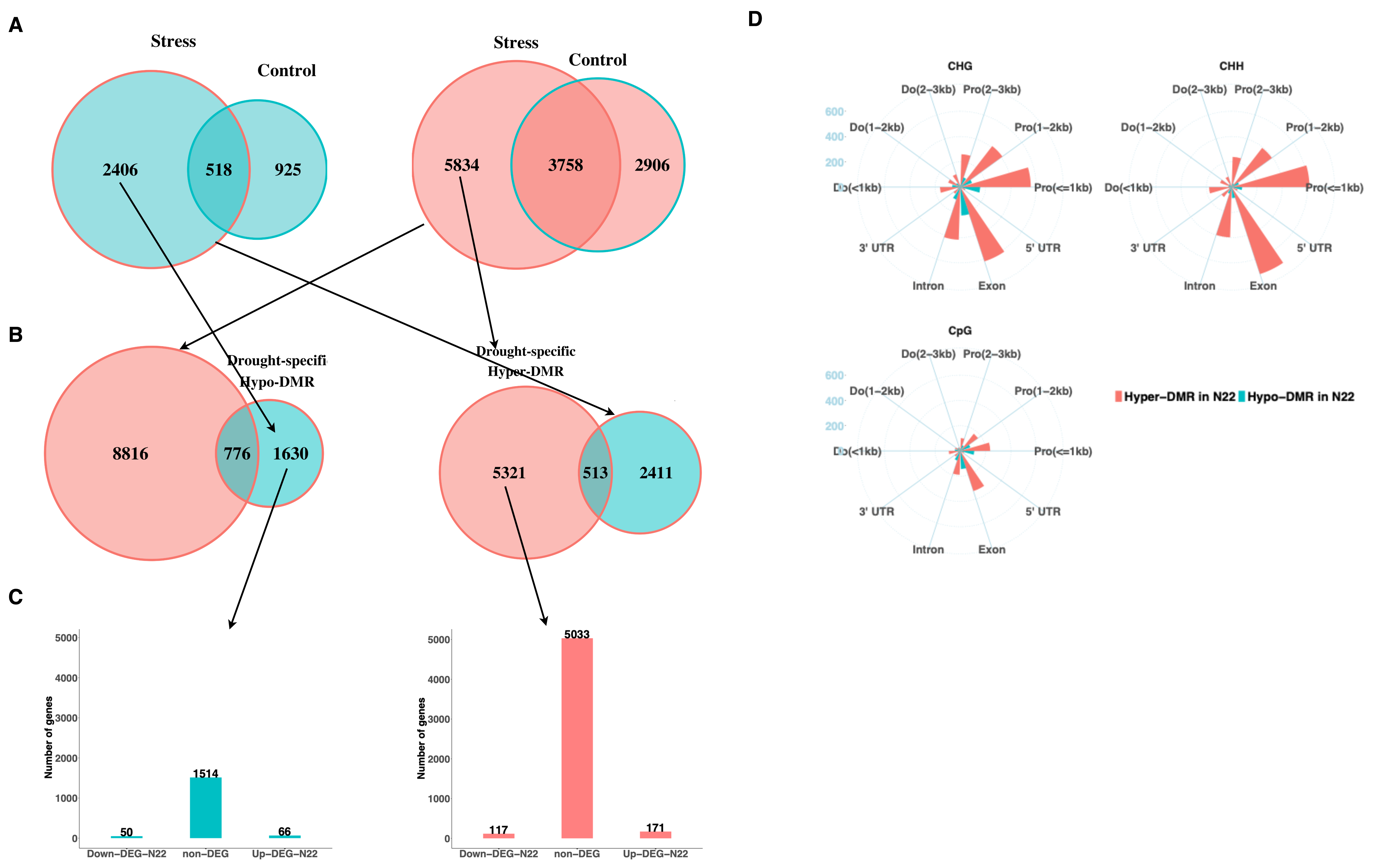


Figure S 11. Genes with drought-specific cDMR in flag leaf. A) total number of genes with hyper- and hypo-cDMR under control and drought stress. B) Genes with drought-specific hyper- and hypo-cDMR. C) Expression status of genes with drought-specific cDMR. Down-DEG-N22 and up-DEG-N22 indicates down-regulated and up-regulated DEGs in N22 compared to IR64, and non-DEG indicated non differentially expressed genes between the two cultivars. D) Distribution pattern of drought-specific hyper and hypo-cDMR across gene elements. Pro – promoter region, Do – Downstream region. Numeric value in the circle represents number of genes. Cyan and red color circumference of circles indicate control and stress. Blue and red color filled circles indicate hyper and hypo-cDMR.
