# Supplementary material for: Genome-wide DNA methylation dynamics at “heading” stage of panicle and flag leaf in contrasting rice cultivars under field drought conditions
Source: Front Plant Sci. 2025 Nov 11;16:1707950. doi: 10.3389/fpls.2025.1707950 (PMC12659711; doi:10.3389/fpls.2025.1707950)
Supplement: Supplementary file 2 [file Table1.docx]

Table S 1. Summary of bisulphite sequencing data summary.

|  | **IR64** | | | | **N22** | | | | | | |
| --- | --- | --- | --- | --- | --- | --- | --- | --- | --- | --- | --- |
|  | **Control** | | **Stress** | | **Control** | | | | **Stress** | | |
|  | **R1** | **R2** | **R1** | **R2** | **R1** | | **R2** | | **R1** | | **R2** |
| **Panicle** | | | | | | | | | | | |
| Total read pairs | 108775050 | 108615320 | 87462910 | 87410674 | 116084504 | | 115997922 | | 81779620 | | 81706468 |
| Mapped reads | 98474279 | 98417706 | 76775560 | 79792363 | 113455508 | | 113353608 | | 79621802 | | 79536488 |
| Uniquely mapped reads | 65882297 | 65890725 | 52166808 | 52217273 | 79869245 | | 79918821 | | 55886299 | | 55874636 |
| Mapping efficiency (%) | 60.57 | 60.66 | 59.64 | 59.74 | 68.80 | | 68.90 | | 68.34 | | 68.38 |
| Genome cytosines (Cs) | 162152322 | 162152322 | 162152322 | 162152322 | 162152322 | | 162152322 | | 162152322 | | 162152322 |
| Cs covered | 144846011 | 145082574 | 144256560 | 144412461 | 145775492 | | 146175221 | | 144214688 | | 144561214 |
| Error rate (%) | 1.44 | 1.44 | 1.44 | 1.44 | 1.44 | | 1.44 | | 1.44 | | 1.44 |
| Total methylated Cs | 13321653 | 13321653 | 11036091 | 11036091 | 14526756 | | 14526756 | | 11835387 | | 11835387 |
| Genome coverage | 16 | 16 | 13 | 13 | 20 | | 20 | | 13 | | 13 |
| **Flag leaf** | | | | | | | | | | | |
| Total read pairs | 97366750 | 97391149 | 83949520 | 83895364 | 28844104 | 28691648 | | 38330148 | | 38338696 | |
| Mapped reads | 90264283 | 90231611 | 82197466 | 82133941 | 24947271 | 24765250 | | 33938161 | | 33743187 | |
| Uniquely mapped reads | 60821572 | 60891261 | 55933508 | 55974025 | 17124076 | 16985199 | | 23465133 | | 23316183 | |
| Mapping efficiency (%) | 62.47 | 62.52 | 66.63 | 66.72 | 59.37 | 59.20 | | 61.22 | | 60.82 | |
| Genome cytosines (Cs) | 162152322 | 162152322 | 162152322 | 162152322 | 162152322 | 162152322 | | 162152322 | | 162152322 | |
| Cs covered | 144996348 | 145259235 | 144087244 | 144488960 | 129399739 | 129474940 | | 135359399 | | 135379319 | |
| Error rate (%) | 1.44 | 1.44 | 1.44 | 1.44 | 1.39 | 1.39 | | 1.39 | | 1.39 | |
| Total methylated Cs | 11985025 | 11985025 | 11271411 | 11271411 | 2365416 | 2365416 | | 4285371 | | 4285371 | |
| Genome coverage | 15 | 15 | 14 | 14 | 4 | 4 | | 6 | | 6 | |

Table S 2. Summary of methylated cytosines under control and drought stress in IR64 and N22. Value in the bracket indicates the percentage of methylated cytosines out of the total cytosines covered (rounded to first digit).

| **Treatment** | **Tissue** | **Cultivar** | **Context** | **Total cytosines covered** | **Methylated cytosine** | **Total methylated cytosines** | **Methylated context out of the total methylated cytosines (%)** |
| --- | --- | --- | --- | --- | --- | --- | --- |
| **Control** | **Panicle** | IR64 | CpG | 27180898 | 6417984 (24) | 13321653  (9) | 48 |
|  |  |  | CHG | 24536211 | 3867928 (16) |  | 29 |
|  |  |  | CHH | 93128902 | 3035741 (3) |  | 23 |
|  |  | N22 | CpG | 27274654 | 7182106 (26) | 14526756 (10) | 49 |
|  |  |  | CHG | 24651342 | 4362850 (18) |  | 30 |
|  |  |  | CHH | 93849496 | 2981800 (3) |  | 21 |
|  | **Flag leaf** | IR64 | CpG | 27144702 | 6125398 (23) | 11985025  (8) | 51 |
|  |  |  | CHG | 24529960 | 3579035 (15) |  | 30 |
|  |  |  | CHH | 93321686 | 2280592 (2) |  | 19 |
|  |  | N22 | CpG | 24563410 | 1200561 (5) | 2365416  (2) | 51 |
|  |  |  | CHG | 22218479 | 782623 (4) |  | 33 |
|  |  |  | CHH | 82617850 | 382232 (1) |  | 16 |
| **Stress** | **Panicle** | IR64 | CpG | 26969076 | 5396594 (20) | 11036091  (8) | 49 |
|  |  |  | CHG | 24408568 | 3258908 (13) |  | 30 |
|  |  |  | CHH | 92878916 | 2380589 (3) |  | 22 |
|  |  | N22 | CpG | 26951964 | 5878606 (22) | 11835387  (8) | 50 |
|  |  |  | CHG | 24405006 | 3635670 (15) |  | 31 |
|  |  |  | CHH | 92857718 | 2321111 (2) |  | 20 |
|  | **Flag leaf** | IR64 | CpG | 26998494 | 5785983 (21) | 11271411  (8) | 51 |
|  |  |  | CHG | 24403464 | 3389949 (14) |  | 30 |
|  |  |  | CHH | 92685286 | 2095479 (2) |  | 19 |
|  |  | N22 | CpG | 25545232 | 2216102 (9) | 4285371  (3) | 52 |
|  |  |  | CHG | 23125840 | 1377810 (6) |  | 32 |
|  |  |  | CHH | 86688327 | 691459 (1) |  | 16 |

Table S 3. Summary of methylated cytosines overlapped between IR64 and N22 under control condition. Value in the bracket represents percentage of the overlapped cytosines out of the total methylated cytosine for the particular context. mC represents methylated cytosine.

| **Tissue** | **CpG** | **CHG** | **CHH** | **Total overlapped mCs** | **Percentage of overlapped out of the total mCs** | |
| --- | --- | --- | --- | --- | --- | --- |
|  |  |  |  |  | **IR64** | **N22** |
| Panicle | 4880594  (52) | 2856086  (31) | 1564472  (17) | 9301152 | 70 | 64 |
| Flag leaf | 1005792  (54) | 623210  (34) | 215639  (12) | 1844641 | 15 | 78 |

Table S 4. Summary of overlapped methylated cytosines between flag leaf and seedling data from a previous study (Garg et al., 2015)

| Cultivar | Treatment | Total overlapped | CpG | CHG | CHH |
| --- | --- | --- | --- | --- | --- |
| IR64 | control | 2314117 | 1214369 | 726351 | 373397 |
| N22 | control | 2756461 | 1425271 | 911403 | 419787 |

Table S 5. Summary table of methylated cytosines that overlapped between panicle and flag leaf in IR64 and N22. mC represents methylated cytosine.

| **Tissue** | **cultivar** | **CpG** | **CHG** | **CHH** | **Total overlapped mC** |
| --- | --- | --- | --- | --- | --- |
| Panicle vs Flag leaf | IR64 | 5378576 | 3093660 | 1520780 | 9993016 |
|  | N22 | 1184074 | 749971 | 246993 | 2181038 |

Table S 6. Methylated cytosines found overlapped between control and drought stress.

| **Tissue** | **Cultivar** | **Overlapped mC (Control and drought stress)** | | | | **Percentage of overlapped mC out of the total mCs found in control (%)** |
| --- | --- | --- | --- | --- | --- | --- |
|  |  | **CpG** | **CHG** | **CHH** | **Total** |  |
| Panicle | IR64 | 4880710 | 2869784 | 1597097 | 9347591 | 70 |
|  | N22 | 5492595 | 3283865 | 1639393 | 10415853 | 72 |
| Flag leaf | IR64 | 5041801 | 2859964 | 1336126 | 9237891 | 77 |
|  | N22 | 957972 | 597509 | 183980 | 1739461 | 74 |

Table S 7. Total number of DI (de-methylated at stress) and DII (de novo methylation at stress) found in IR64 and N22.

| Tissue | Cultivar | **DI** | **DII** | **DII out of the total mC at control (%)** | **DIII out of the total mC at stress (%)** |
| --- | --- | --- | --- | --- | --- |
| Panicle | IR64 | 3974062 | 1688500 | 30 | 15 |
|  | N22 | 4110903 | 1419534 | 28 | 12 |
| Flag leaf | IR64 | 2747134 | 2033520 | 23 | 18 |
|  | N22 | 625955 | 2545910 | 26 | 59 |

Table S 8. Number of overlapped genes between cultivars that associate with DI (de-methylated cytosines) and DII (de novo methylated cytosines).

| **Type** | **Tissue** | **Expression category** | **Total gene in IR64** | **Total gene in N22** | **Overlapped gene** | **Genes in N22 overlapped with IR64 (%)** |
| --- | --- | --- | --- | --- | --- | --- |
| **DI** | panicle | silent | 31057 | 31397 | 28753 | 92 |
|  |  | low | 10443 | 11317 | 7181 | 63 |
|  |  | medium | 6083 | 5687 | 3128 | 55 |
|  |  | high | 6239 | 5662 | 4403 | 78 |
|  | flag leaf | silent | 30805 | 24041 | 22342 | 93 |
|  |  | low | 11258 | 8073 | 5529 | 68 |
|  |  | medium | 5721 | 4125 | 2303 | 56 |
|  |  | high | 5681 | 4262 | 3238 | 76 |
| **DII** | panicle | silent | 30408 | 30004 | 27246 | 91 |
|  |  | low | 10315 | 10813 | 6832 | 63 |
|  |  | medium | 6010 | 5468 | 2983 | 54 |
|  |  | high | 6157 | 5371 | 4157 | 77 |
|  | flag leaf | silent | 30318 | 28570 | 26133 | 91 |
|  |  | low | 11093 | 10121 | 6884 | 68 |
|  |  | medium | 5638 | 5100 | 2855 | 56 |
|  |  | high | 5559 | 5015 | 3726 | 74 |

Table S 9. Drought-induce DMR found in all the tissues of IR64 and N22.

| **Tissue** | **Cultivar** | **context** | **count** | **Total DMRs** |
| --- | --- | --- | --- | --- |
| Panicle | IR64 | CpG | 940 | 9929 |
|  |  | CHG | 2182 |  |
|  |  | CHH | 6807 |  |
|  | N22 | CpG | 649 | 9199 |
|  |  | CHG | 2803 |  |
|  |  | CHH | 5747 |  |
| Flag leaf | IR64 | CpG | 738 | 4639 |
|  |  | CHG | 1520 |  |
|  |  | CHH | 2381 |  |
|  | N22 | CpG | 145 | 673 |
|  |  | CHG | 103 |  |
|  |  | CHH | 425 |  |

Table S 10. Number of DEG whose expressions were negatively correlated with the methylation level of dDMR. dDMR found within 3kb upstream to 3kb downstream regions of gene were considered for this analysis.

| **Tissue** | **Cutlivar** | **DMR type** | **DEG status** | **Count of DEG** |
| --- | --- | --- | --- | --- |
| **Panicle** | IR64 | Hyper-DMR | Down-DEG | 79 |
|  |  | Hypo-DMR | Up-DEG | 36 |
|  | N22 | Hyper-DMR | Down-DEG | 269 |
|  |  | Hypo-DMR | Up-DEG | 24 |
| **Flag leaf** | IR64 | Hyper-DMR | Down-DEG | 32 |
|  |  | Hypo-DMR | Up-DEG | 33 |
|  | N22 | Hyper-DMR | Down-DEG | 1 |
|  |  | Hypo-DMR | Up-DEG | 9 |
